# Functional diversity of avian communities increases with canopy height: From individual behavior to continental‐scale patterns

**DOI:** 10.1002/ece3.7952

**Published:** 2021-07-27

**Authors:** Vladimír Remeš, Eva Remešová, Nicholas R. Friedman, Beata Matysioková, Lucia Rubáčová

**Affiliations:** ^1^ Department of Zoology and Laboratory of Ornithology Faculty of Science Palacky University Olomouc Czech Republic; ^2^ Department of Ecology Faculty of Science Charles University Prague Czech Republic; ^3^ Environmental Informatics Section Okinawa Institute of Science and Technology Graduate University Onna‐son Japan; ^4^ Department of Zoology Faculty of Natural Science Comenius University Bratislava Slovakia

**Keywords:** birds, foraging behavior, functional diversity, resource partitioning, species richness, vegetation complexity

## Abstract

Vegetation complexity is an important predictor of animal species diversity. Specifically, taller vegetation should provide more potential ecological niches and thus harbor communities with higher species richness and functional diversity (FD). Resource use behavior is an especially important functional trait because it links species to their resource base with direct relevance to niche partitioning. However, it is unclear how exactly the diversity of resource use behavior changes with vegetation complexity. To address this question, we studied avian FD in relation to vegetation complexity along a continental‐scale vegetation gradient. We quantified foraging behavior of passerine birds in terms of foraging method and substrate use at 21 sites (63 transects) spanning 3,000 km of woodlands and forests in Australia. We also quantified vegetation structure on 630 sampling points at the same sites. Additionally, we measured morphological traits for all 111 observed species in museum collections. We calculated individual‐based, abundance‐weighted FD in morphology and foraging behavior and related it to species richness and vegetation complexity (indexed by canopy height) using structural equation modeling, rarefaction analyses, and distance‐based metrics. FD of morphology and foraging methods was best predicted by species richness. However, FD of substrate use was best predicted by canopy height (ranging 10–30 m), but only when substrates were categorized with fine resolution (17 categories), not when categorized coarsely (8 categories). These results suggest that, first, FD might increase with vegetation complexity independently of species richness, but whether it does so depends on the studied functional trait. Second, patterns found might be shaped by how finely we categorize functional traits. More complex vegetation provided larger "ecological space" with more resources, allowing the coexistence of more species with disproportionately more diverse foraging substrate use. We suggest that the latter pattern was driven by nonrandom accumulation of functionally distinct species with increasing canopy height.

## Graphical abstract

Species diversity should increase with vegetation complexity, because more complex vegetation provides larger “ecological space” with more potential niches. We demonstrate increasing diversity of resource use with increasing canopy height in Australian passerines. However, we also show that this relationship critically depends on trait selection and resolution.

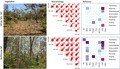

## 1 INTRODUCTION

Habitat heterogeneity has been identified as an important predictor of animal species diversity (Tews et al., 2004). Vegetation complexity is a kind of habitat heterogeneity expressed for example as the number of vegetation layers, the amount of foliage, or canopy height. The vegetation metric used should reflect the amount of available resources and the number of potential ecological niches. It has been shown that species richness increases with vegetation complexity, quantified in different ways, in both insects (Scherber et al., 2010) and vertebrates (Jankowski et al., 2013; Jiménez‐Alfaro et al., 2016; Sam et al., 2019; Santillán et al., 2020). More specifically, taller vegetation is thought to provide more resources and potential ecological niches where more species can coexist (MacArthur & MacArthur, 1961; Willson, 1974). Accordingly, vertebrate species richness has been shown to increase along vegetation succession series (Blondel & Farré, 1988; Reif et al., 2013) and along gradients of vegetation height on continental (Culbert et al., 2013; Gouveia et al., 2014; Ilsøe et al., 2017; James & Wamer, 1982; Remeš & Harmáčková, 2018) and global scales (Feng et al., 2020; but see Coops et al., 2018; Roll et al., 2015). However, besides species richness, analyzing species' ecological functions can provide unparalleled insights into processes organizing communities (Cadotte & Davies, 2016; Hutchinson, 1959; Lack, 1971; McGill et al., 2006), especially along environmental gradients (Oliveira & Scheffers, 2019; Vollstädt et al., 2017). For example, behavior is a prime example of a functional trait with great ecological importance, because it provides a critical link between the organism and its environment and has great consequences for the individual's fitness.

Resource use behavior is especially important because it links each species to its resource base, and indeed, evolutionary divergence in resource use behavior has facilitated spectacular adaptive radiations (Friedman et al., 2019; Jønsson et al., 2012; Ronco et al., 2021). Studies of foraging behavior in local communities have documented fine‐scale partitioning of ecological niches and putative mechanisms of species coexistence (Holmes et al., 1979; MacArthur, 1958; Morrison et al., 1990; Remešová et al., 2020). On the other hand, global and continental‐scale studies of food and foraging provided insights into spatial gradients of functional diversity and broadscale species co‐occurrence (Barnagaud et al., 2014; Miller et al., 2017; Schumm et al., 2019; Stevens et al., 2003; Terborgh & Robinson, 1986). We suggest that there is a great potential in combining these two approaches, namely studying functional diversity of replicated local communities across large‐scale environmental gradients. This approach would allow for detailed partitioning of how species richness and functional diversity change with vegetation complexity. Yet, few studies exist that have integrated both local and continental scales. Moreover, those that did compared communities in the same environments, for example, deserts or mature forests (Korňan et al., 2013; Pianka, 1986). Consequently, they were not able to capitalize on insights provided by environmental gradients (Tylianakis & Morris, 2017).

Several problems must be overcome when studying species richness and functional diversity across spatial scales and along large gradients of vegetation complexity. First, most global and continental‐scale studies are based on a spatial grain of geographical cells with the resolution of 100 × 100 km (e.g., Barnagaud et al., 2014; Feng et al., 2020; Remeš & Harmáčková, 2018; Schumm et al., 2019). However, species interactions occur locally. Second, these studies typically used species pseudo‐occurrences inferred from global range maps, without actual, ground‐truthed occurrences and may thus misrepresent co‐occurrence. Third, species–abundance distributions are highly skewed (e.g., Alroy, 2015), whereas both range maps and pure occurrence data assume uniformity. Thus, working with occurrences and not taking into account actual species abundances provides a biased picture of the functional diversity of communities. Fourth, studies often used subjective a priori approach to functional trait assignment (Wilson, 1999) by using coarse species functional categorizations available in global datasets (Feng et al., 2020; Pellissier et al., 2018; Schumm et al., 2019; Wilman et al., 2014). This approach neglects environmental context, and plasticity and intraspecific variation in behavior, which can jeopardize inferring ecological processes (Allgeier et al., 2017; Ross et al., 2017; Violle et al., 2012). Moreover, coarse ecological and behavioral categories can limit the validity of insight into the functional diversity of communities (Maire et al., 2015; Rosado et al., 2016). The remedy to these problems is to study functional diversity of local communities along large‐scale gradients in vegetation complexity. This approach prevents many of the outlined problems by simultaneously providing detailed data on diversity, extensive spatial replication, and strong local environmental context.

**FIGURE 1:**
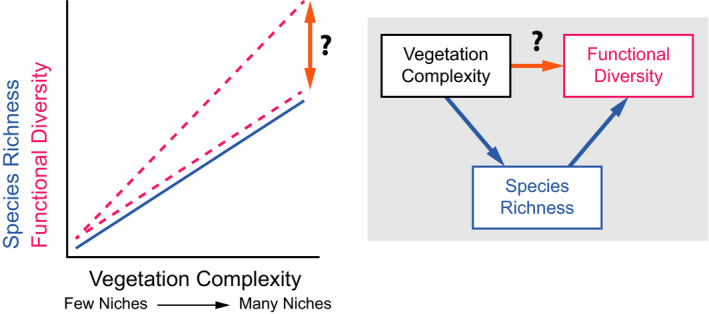
Conceptual depiction of relationships between vegetation complexity, species richness (SR), and functional diversity (FD). SR has been demonstrated to increase with vegetation complexity (full blue line). Due to a sampling effect, FD should increase with SR (if not adjusted for SR statistically). However, it is unclear whether FD would increase at the same rate as SR or faster (hatched pink lines). In the latter case, there would be a direct, additive link between vegetation complexity and FD (orange paths with question marks). Exact rate of increase of FD with vegetation complexity and SR can differ between functional traits and between different resolutions of the same trait, which is an underexplored question of ecology

**FIGURE 2:**
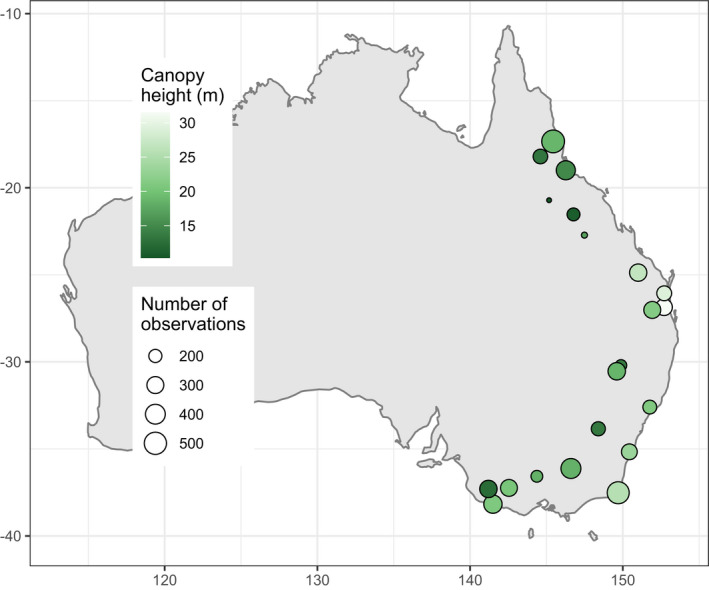
Map of Australia showing the location of our 21 study sites. The latitudinal span of the sites is ca. 2,300 km, while the span along the coast is ca. 3,000 km. "Number of observations" is the number of foraging records obtained on each site. For the names of individual sites, see Figure S1

Here, we analyzed resource use behavior at individual level in local communities while comparing replicated communities along a continental‐scale gradient in vegetation complexity. More specifically, we asked whether and how species richness, morphological diversity, and foraging behavior of local avian communities change along a continental‐scale gradient in vegetation complexity (Figure 1). We studied communities of passerine birds on 21 sites (63 transects) in woodlands and forests of eastern Australia (Figure 2). We accounted for the problems we outlined above by studying communities on a small spatial grain and using abundance‐weighted, individual‐based functional diversity indices of both morphology and foraging behavior; we also provided community‐level environmental context by locally sampling vegetation. We expected more complex vegetation to provide more and diverse foraging substrates and thus to harbor more species with more diverse morphologies and foraging strategies (Figure 1).

## 2 METHODS

### 2.1 Definitions

We define *functional trait* as any trait measurable at an individual level with a demonstrated or strongly implicated link to fitness. Thus, we consider foraging behavior (methods and substrates) and morphology as functional traits, even though foraging behavior can be defined only in relation to the surrounding environment (i.e., not measurable on the isolated individual). We define *functional diversity* as an index calculated using measured values of functional traits. Here, we calculate it using individual‐based, abundance‐weighted measurements and observations and aggregate it at the level of local communities. For each functional trait, we use the functional diversity index recommended in literature (see below). We define *vegetation complexity* as any index of vegetation structure that has a potential to predict the amount of available resources and the number of potential ecological niches. Here, we use several indices, show that they are intercorrelated, and use one of them for downstream analyses (see below).

### 2.2 Study design and data collection

We worked at 21 sites in eucalypt woodlands and forests in eastern Australia (Figure 2; for the names of the sites, see Figure S1) from September to December in 2016 and 2017 (Table S1). At each site, we delimited three transects; each transect was 2 km long and 50 m wide (10 ha). We placed the transects such that they were representative of local vegetation.

To quantify vegetation structure, we placed 10 points (200 m apart) along each transect (*n* = 630 sampling points in total). At each point, we delimited a semi‐circle with the radius of 25 m (area ca. 0.1 ha or 1,000 m^2^) and recorded vegetation cover in five *height strata* (0–1 m, 1.1–2.0 m, 2.1–5 m, 5.1–10 m, >10 m) and vegetation cover and height of four *vegetation strata* (herbaceous, shrub, subcanopy, and canopy). Height strata were delimited by a priori selected height bands, while vegetation strata were determined by major vertical vegetation layers typical of woodlands and forests. Thus, for vegetation strata, besides cover we also had to measure the height of individual strata. Vegetation cover was estimated by eye on a scale ranging from 0 (no vegetation) to 10 (fully covered), and vegetation height was measured by a laser rangefinder (Nikon Forestry).

We recorded foraging behavior of all passerines (Passeriformes). Each transect was walked twice in the morning for 4 hr by two observers with 0–3 days between the two censuses. Once we located a bird, we recorded a maximum of three events of procuring or attempting to procure food (*prey attack*; mean = 2.25 attacks per individual bird, *n* = 2,624 individual birds). We were not able to record three prey attacks for every individual, because it might have stopped foraging or flown away. For each prey attack, we recorded bird species, foraging method and substrate, distance from the plant stem, and foliage density around the foraging bird (Table 1). More details on field methods are available in Appendix S1.

**TABLE 1:** Description of foraging behaviors recorded for each prey attack

Main category	Subcategory	Definition
Foraging methods (*n* = 8)		
Gleaning		Moving on/through the substrate and taking prey from its surface; prey is taken while the bird is on the substrate.
Hang‐gleaning		Gleaning while the bird is hanging upside‐down.
Snatching		Moving on/through the substrate and making short flights to take the prey from nearby substrates.
Hover‐snatching		Snatching while the bird stays in the air (hovers) when taking the prey from a substrate.
Probing		Extracting food from/within thick or deep substrate, such as soil, litter, or flowers.
Manipulation		Includes variety of methods such as scratching, digging, and tearing to expose the prey.
Pouncing		Direct flight from a perch to the site where the prey is taken (usually ground), whereby the bird lands and takes the prey.
Flycatching		Flying from a substrate to take a flying prey, whereby both the foraging bird and prey are in the air.

Main substrates (*n* = 8)	Fine substrates (*n* = 17)	
Ground	Bare ground	No cover on the ground.
	Leaf litter	Ground covered with leaf litter.
	Grass	Ground covered by grass or other low vegetation.
Leaf	Small leaf	Any dimension of the leaf below ca. 10 cm.
	Large leaf	Larger than ca. 10 cm.
Bark	Twig	Bears leaves at the end of a branch.
	Small branch	Diameter below ca. 10 cm.
	Large branch	Diameter over ca. 10 cm.
	Trunk	Vertical stem supporting a shrub or a tree.
Bud	Bud	Unopened leaf or flower.
Flower	Flower	Any size or type of flower.
Fruit	Fruit	Includes dry and fleshy fruits.
Air	Above trees	Above canopy but not high in the sky.
	Between trees	Between canopies of shrubs and trees.
	Within trees	Within a canopy.
	Over ground	Below the height of ca. 1 m.
Other	Other	Special substrates, for example, spider webs.

Distance from stem (*n* = 4)		
Directly on stem		Bird touches the vertical stem.
Inner half of crown		Foraging within the inner 50% of the crown volume.
Outer half of crown		Foraging within the outer 50% of the crown volume.
Edge of crown		Bird moves along the shrub or tree crown diameter.

Foliage density (*n* = 3)		
Low		Bird easily visible; includes zero foliage.
Medium		Intermediate.
High		Bird difficult to see.

To obtain morphological measurements for species we studied, we visited collections of Natural History Museum in Tring (UK), American Museum of Natural History in New York City (USA), and Australian National Wildlife Collection in Canberra (Australia). Here, we measured beak length, width and depth, wing length, tarsus length, and tail length in at least three males and three females per species. We measured beak, wing, and tarsus dimensions with digital calipers to the nearest 0.1 mm. We measured beak length from its tip to the edge of the skull, and beak width and depth at the distal edges of the nostrils. We measured tail length with a paper ruler to the nearest 0.5 mm by inserting the ruler between tail feathers and under‐tail coverts and reading the length of the tail at its tip. These measures have been commonly used in avian functional ecology (e.g., Ricklefs, 2012). We took body mass from the Handbook of Australian, New Zealand, and Antarctic Birds (Higgins et al., 2006).

### 2.3 Data analyses

#### 2.3.1 Vegetation complexity

We constructed vegetation profiles of our 21 study sites (each site based on 30 vegetation sampling plots) showing vegetation cover in individual strata (Figure S2). We then calculated four indices of vegetation complexity for every site: (a) summed vegetation cover of height strata (i.e., within a priori delimited height bands; see above), (b) summed vegetation cover of vegetation strata (i.e., herbaceous, shrub, subcanopy, and canopy; see above), (c) average canopy height (m), and (d) canopy cover (%). These four variables were highly correlated (*r* > 0.77; except the correlation between canopy height and canopy coverage where *r* = 0.52; Figure S3). This collinearity was confirmed by a PCA run on all vegetation characteristics (Figure S4). The first PC axis explained 48% of variability, correlated positively with most vegetation characteristics (Figure S4), and correlated also well with other vegetation metrics (*r* > 0.72; Figure S3). We used canopy height in further analyses because it reliably expressed overall vegetation volume (Figure S3) and has been recently used in many studies of animal diversity (e.g., Coops et al., 2018; Feng et al., 2020; Gouveia et al., 2014; Remeš & Harmáčková, 2018; Roll et al., 2015).

#### 2.3.2 Morphological and behavioral diversity

We quantified functional diversity (FD) of avian assemblages at our 21 sites in terms of morphology and foraging behavior. To quantify site‐specific morphological FD, we used the hypervolume method of Blonder et al. (2018) applied to seven morphological traits. It is a new algorithm for delineating the boundaries and probability density within n‐dimensional hypervolumes and is implemented in the *hypervolume* package for R (Blonder & Harris, 2019). Unlike traditional convex hull volume (Cornwell et al., 2006), this method has the advantage of allowing for holes in morphological hyperspace (Blonder, 2016). Moreover, due to its probabilistic nature, and again compared with convex hull volume, it is not so sensitive to outlying data points (Blonder et al., 2018). It thus brings more robust approach for delineating the shape and density of n‐dimensional hypervolumes. Moreover, it improves further on the precision of estimating hypervolumes by using abundance‐weighted data. The total volume of the 7‐dimensional morphological hyperspace was thus a robust estimate of morphological FD at the level of our study sites. In terms of computational choices, we used the "silverman" method when calculating bandwidth vector from data using the "estimate_bandwidth" function. Then, we constructed a hypervolume by using a Gaussian kernel density estimate (the "hypervolume_gaussian" function). This method is preferable because hypervolume estimates are not biased by extreme values (it provides a "loose wrap" to the data) and is recommended for most functional diversity applications (Blonder et al., 2018). We used default settings for the number of random points to be evaluated.

We used multiple FD indices and functional traits to calculate behavioral diversity at our 21 sites. We used (a) several indices of FD recommended by Schleuter et al. (2010) for categorical traits, and (b) several indices of niche breadth (Krebs, 1999). More specifically, we calculated functional richness (IN 1.2. in table 1 of Schleuter et al., 2010), functional evenness (IN 2.1), functional divergence (IN 3.3), and indices of Levins, Simpson, and Shannon (Krebs, 1999). All these indices correlated positively, and thus, we chose only the Shannon index for all subsequent analyses. The reasons were twofold. First, we used rarefaction to account for different numbers of individuals sampled across sites for both species richness and FD (see below), and rarefaction is available only for the Shannon and Simpson diversity (Hsieh et al., 2016). Second, Shannon index correlated most closely with all other indices (Appendix S2).

We defined several combinations of behavioral traits recorded in the field (Table 1, Appendix S3) to assess the sensitivity of our results to methodological choices. Using all these combinations, we calculated all FD indices and assessed their sensitivity to how finely we categorized foraging behavior. Most of the correlations between different combinations of behavioral traits were high for all FD indices (Appendix S3). For further analyses, we used foraging methods (*n* = 8 categories), a crossed matrix of foraging methods and main foraging substrates (hereafter “foraging‐substrate combinations”; *n* = 50), and foraging substrates with three levels of resolution: main substrates (*n* = 8), fine substrates (*n* = 17), and all substrates (*n* = 24; including fine substrates, distance from stem, and foliage density, see Table 1). Further details are available in Appendix S3.

### 2.4 Statistical analyses

We checked that our sampling of species richness and behavioral functional diversity across sites was sufficient by calculating sample coverage (Chao & Jost, 2012) using rarefaction analyses in the *iNEXT* package for R (Hsieh et al., 2016). Across sites, sample coverage was >0.88 for species richness, >0.99 for foraging methods, >0.99 for foraging substrates, and >0.97 for the foraging‐substrate combinations. We were thus confident that inadequate sampling was not an issue, and our estimates of FD were reliable (Pakeman, 2014).

We aimed to analyze both direct and indirect (via species richness) relationships between FD and vegetation complexity (Figure 1). However, we had to account for the fact that total densities (i.e., the total number of individuals per transect; Gotelli, 2008) differed among sites and, in fact, increased with canopy height (*r* = 0.43). We included total density only as a nuisance parameter and did not discuss it further. We used three analytical approaches. First, we used structural equation modeling (SEM) where FD was the variable to be explained and three other variables (Figure 1) were linked in hypothesized causal relationships in the SEM (Shipley, 2009). To fit the SEM, we used a piecewise approach in which the causal relationships were statistically defined and evaluated as mutually interconnected equations using the *piecewiseSEM* package for R (Lefcheck, 2016). Specifically, we used the following three linear equation formulas:

1. Species richness ~log10(Canopy height) + Total density
2. Total density ~log10(Canopy height)
3. FD ~log10(Canopy height) + Species richness + Total density.

We fitted these equations using the “gls” function in the *nlme* package for R (Pinheiro et al., 2021) and checked for any potential spatial autocorrelation in residuals by using AIC. These three equations were united into a single structural model using the "psem" function of the *piecewiseSEM* package (Lefcheck, 2016). We then extracted standardized regression coefficients and their statistical significance from the resulting R object using the "summary" function. We did not evaluate the overall model fit, as our aim was not model selection but rather parameter estimation.

Second, we calculated the expected species richness and behavioral FD (i.e., FD of foraging behavior) for a standardized total density using individual‐based rarefaction. We used the “estimateD” function in the *iNEXT* package for R (Hsieh et al., 2016). Total density varied from 49 to 220 individuals across sites. The maximum recommended extrapolation extent is to twice the number of individuals (Chao et al., 2014). We thus standardized species richness to 100 individuals. The number of foraging records varied from 112 to 522 (because we recorded up to three foraging events per individual, see above). We standardized behavioral FD to 225 individuals. Rarefaction was not available for the morphological hypervolume‐based FD. Third, we calculated distance‐based measures between pairs of sites and organized them into distance matrices. We calculated distance matrices using (a) Euclidean distances on vegetation characteristics, (b) Bray–Curtis distances on foraging behavior categories (both calculated in the *vegan* package for R, Oksanen et al., 2020), and (c) total Sørensen beta diversity on species abundances (calculated in the *BAT* package for R; Cardoso et al., 2018; see Appendix S4 for justification). For analyses, we used multiple regressions on distance matrices in the *ecodist* package for R (Goslee & Urban, 2007). A distance matrix was not available for the morphological hypervolume‐based FD. We calculated the nestedness and turnover components of beta diversity in the *BAT* package (Cardoso et al., 2018).

Many characteristics of vegetation and species diversity often correlate with latitude. However, Australia has a unique east–west climatic gradient produced by aridification of central Australia (Byrne et al., 2011). This led to the absence of a latitudinal gradient in species richness, FD (Remeš & Harmáčková, 2018), and vegetation complexity (Appendix S4). We thus did not include latitude into our final models. However, we made sure that it could not confound our analyses (Appendix S4). On the other hand, geographical distance is usually an important predictor of beta diversity (Legendre et al., 2005), and thus, we included geographical distance as a covariate into our analyses of distance matrices.

## 3 RESULTS

**FIGURE 3:**
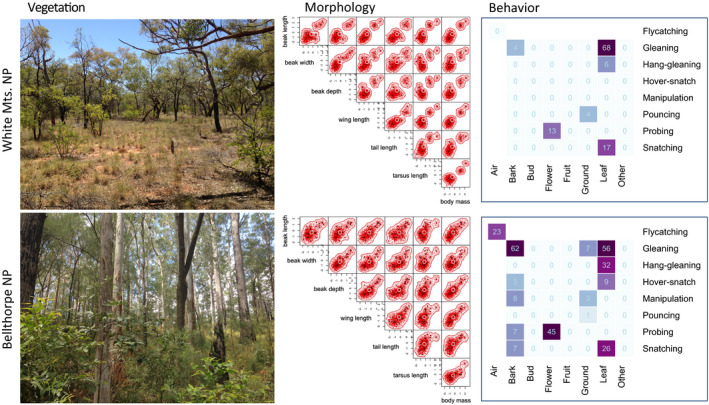
Patterns in vegetation complexity and morphological and behavioral functional diversity. White Mts. and Bellthorpe National Parks (see Figure S1 for locations) provide two extremes of vegetation complexity across our 21 sites in eastern Australia. Vegetation was simple and short in White Mts. NP (mean canopy height was 10.3 m), while it was higher in Bellthorpe NP (canopy 31.5 m; see Figure S2 for complete vegetation profiles of all sites). Morphological functional diversity was smaller in White Mts. than in Bellthorpe, as evidenced by smaller volume of the morphological hyperspace (*n* = 7 traits; see Methods). Each subpanel of the morphological panels shows the relationship between two traits and the projection of the 7‐dimensional hyperspace into the plane. The red area is proportional to the morphological space defined by these two traits that were filled by individuals recorded on the site. Similarly, behavioral functional diversity was smaller in White Mts. than in Bellthorpe, as evidenced by lower occupancy and evenness of foraging‐substrate combinations in White Mts. (*n* = 6 combinations) than in Bellthorpe (*n* = 14). Numbers in foraging‐substrate combinations denote the number of observations, and higher numbers are emphasized by darker colors. Zeroes mean that the combination was not observed, while blank space denotes implausible combinations (e.g., “flycatching on the ground”)

**FIGURE 4:**
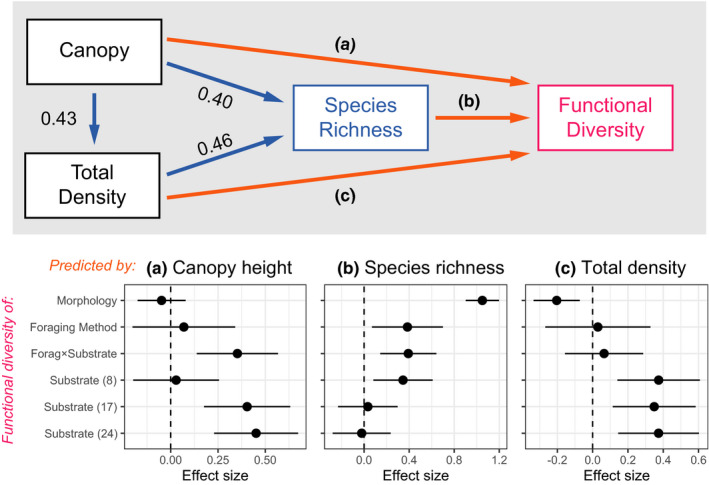
Path models explaining functional diversity in morphology and foraging behavior. Standardized effect sizes are depicted along blue paths; they do not change between models. Effect sizes for different functional traits used to calculate functional diversity change between the models and are depicted (estimate ±1*SE*) in the forest plots (the letter codes along violet paths and on top of the forest plot panels link the two graphs). Functional traits used to calculate functional diversity were following (see Methods): morphology (morphospace hypervolume), foraging method (*n* = 8 categories), foraging‐substrate combinations ("Forag×Substrate," *n* = 50), main substrates ("Substrate (8)," *n* = 8), fine substrates ("Substrate (17)," *n* = 17), and all substrates ("Substrate (24)," *n* = 24)

Functional diversity of morphology and foraging methods and substrates was higher on sites with higher canopy than on sites with lower canopy (Figure 3). However, drivers of these relationships differed between functional traits. Morphological functional diversity increased greatly with species richness, while the direct effect of canopy height was negligible (Figure 4). The situation was similar for foraging methods and the main substrates used for foraging, although the effect of species richness was much weaker (Figure 4). In foraging‐substrate combinations, the effect sizes of species richness and canopy height were comparable (Figure 4). Finally, in both finely categorized and all foraging substrates, canopy height was the main driver of behavioral functional diversity, while the effect of species richness was negligible (Figure 4, detailed statistics in Appendix S5).

**FIGURE 5:**
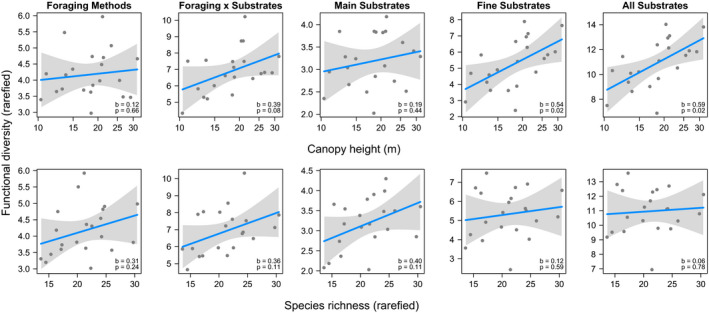
Rarefied estimates of behavioral functional diversity (Shannon index) in relation to canopy height and species richness. Within each panel, standardized effect size ("b") and the associated *p*‐value ("*p*") are given. Functional diversity was resampled for 225 foraging records, while species richness was resampled for 100 individuals. The gray area is the 95% confidence band around the linear regression fit (in blue). Canopy height was log10‐transformed

Analyses of estimates resampled to a standardized number of observations confirmed these insights. Functional diversity of foraging methods and main foraging substrates increased more steeply with species richness than with canopy height, although none of these effects were statistically significant (Figure 5). On the other hand, functional diversity of both fine and all foraging substrates increased greatly and statistically significantly with canopy height, while species richness had virtually no effect (Figure 5). The effect sizes of species richness and canopy height were of similar magnitude in foraging‐substrate combinations (Figure 5, detailed statistics in Appendix S6). Using distance matrices, we tested whether sites with more different vegetation structure and species composition were also further apart in behavioral functional diversity space. Distances in behavioral functional diversity space significantly increased with differences in both vegetation structure and species composition between sites, with the exception of the vegetation effect on main foraging substrates; geographic distance had always negligible effects (Table 2, details in Appendix S7).

**TABLE 2:** Correlations between distance‐based metrics. Correlation coefficients (p‐value) are given for relationships between distance matrices of functional traits and distance matrices of vegetation structure, species composition dissimilarity (beta diversity), and geographic distance (in km). *p* and *R* ^2^ values are from the multiple regression on distance matrices analyses fit in the *ecodist* package for R (Goslee & Urban, 2007)

Predictor	Foraging methods	Forag x Substrate	Main Substrates	Fine Substrates	All Substrates
	(*R* ^2^ = 0.19)	(*R* ^2^ = 0.18)	(*R* ^2^ = 0.13)	(*R* ^2^ = 0.30)	(*R* ^2^ = 0.31)
Vegetation	0.039 (0.021)	0.032 (0.046)	0.003 (0.843)	0.042 (0.018)	0.028 (0.016)
Beta diversity	0.173 (0.016)	0.199 (0.010)	0.219 (0.006)	0.307 (0.001)	0.209 (0.002)
Distance	−0.001 (0.404)	−0.001 (0.522)	−0.001 (0.184)	−0.001 (0.209)	−0.001 (0.188)

We tried and evaluated two potential explanations of the patterns described above. First, each species might have used more foraging substrates in more complex vegetation. We thus tested the within‐species relationship between the functional diversity in substrate use and canopy height, controlled for the number of foraging records per species per site. We did this only for the Brown Thornbill (*Acanthiza pusilla*, *n* = 263 foraging records) and the Yellow‐faced Honeyeater (*Caligavis chrysops*, *n* = 193), the two species with the highest number of foraging records available. For the Brown Thornbill, functional diversity of fine substrate use increased with the number of foraging records (standardized effect size, ES = 0.67, *p* = .017), while the relationship with canopy height was not significant (ES = 0.01, *p* = .979). For the Yellow‐faced Honeyeater, neither canopy height (ES < 0.01, *p* = .987) nor the number of foraging records (ES = 0.20, *p* = .473) significantly predicted functional diversity in fine substrate use (details in Appendix S8). Second, comparison of nestedness and turnover of substrate use versus species composition might shed light on the patterns of accumulation of ecological functions versus species. The accumulation of fine substrates with canopy height was highly nested (nestedness = 0.59, turnover = 0.41). This means that certain substrates were used in almost all sites, while others were used only in sites with high canopy (visualized in Appendix S9). On the other hand, species composition had higher turnover (0.75) than nestedness (0.25). This means that none of the species was detected across all sites and species composition was shaped by a biogeographic turnover of avifaunas (visualized in Appendix S9).

## 4 DISCUSSION

We studied species richness and functional diversity of avian communities along a gradient of canopy height in woodlands and forests of eastern Australia. Both species richness and individual‐based functional diversity of communities increased with canopy height (an index of vegetation complexity). However, the drivers of functional diversity for individual functional traits differed. While functional diversity in morphology and foraging methods was best predicted by species richness, functional diversity of fine substrates used for foraging was best predicted by canopy height itself (Figure 4). These results suggest that, first, functional diversity might increase with vegetation complexity independently of species richness, but whether it does so depends on the studied functional trait. Second, patterns found might be affected by how finely we categorize functional traits (compare effect sizes for main, fine, and all foraging substrates in Figure 4). Overall, sites with more complex vegetation provided a larger "ecological space" with more resources, allowing the coexistence of more species with disproportionately more diverse foraging substrate use.

The additional resources provided by taller vegetation may increase functional diversity of local communities by allowing the coexistence of species characterized by more diverse ecological strategies (Aguirre‐Gutiérrez et al., 2017; Oliveira & Scheffers, 2019). For example, tall forest vegetation provides more foraging substrate for bark‐foraging species of birds and, accordingly, the proportion of bark‐foraging events increased with increasing canopy height across our study sites (Remešová et al., 2020). On the other hand, in a study of the mulga *Acacia aneura* habitat with canopy cover less than 20%, songbirds foraged mostly from the ground (63.8% of foraging records; Recher & Davis, 1997). This was probably caused by the relative paucity of taller vegetation compared to eucalypt woodlands and forests and by the fact that mulga has low canopy cover and often grows as a monoculture with little or no mid‐story and often no understory. However, these examples concern foraging substrate use. Whether functional diversity increased with canopy height independently of species richness differed across different functional traits and their resolution. Species richness alone was sufficient to explain morphological functional diversity. This is probably not surprising, as morphology is usually more tightly linked to phylogeny than behavior (Blomberg et al., 2003). Functional diversity of foraging methods and main foraging substrates (coarsely categorized) were also better predicted by species richness than by canopy height. The opposite was true for functional diversity of foraging substrates categorized with higher resolution (into many categories). The effects of species richness and canopy height were of similar magnitude in functional diversity of foraging‐substrate combinations. Thus, trait selection (foraging method vs. substrate) and trait category resolution seem to independently shape the effect of vegetation complexity on functional diversity.

We see two implications of our results. First, bird species partition ecological space more in terms of foraging substrates than foraging methods (Harmáčková et al., 2019). An analogous phenomenon seems to contribute to higher functional diversity in tropical as compared to temperate forests (Schumm et al., 2019). For example, tropical forests harbor many species eating large fruits and foraging on dead leaves, vines, lianas, etc.—substrates not available in temperate forests (Bell, 1983; Rosenberg, 1997; Sillett et al., 1997; Terborgh, 1980). Second, partitioning of resources becomes apparent only when we recognize enough categories of functional traits. Of course, it remains unclear how many categories are "enough," but this problem may have caused mixed results of previous studies relating functional diversity to vegetation complexity (Feng et al., 2020; Remeš & Harmáčková, 2018; Vollstädt et al., 2017). General ecological implication is that using only a handful of functional trait categories can lead to missing niche axes critical for the detection of resource partitioning and species coexistence in natural communities.

Two mutually nonexclusive processes might explain increasing functional diversity of foraging substrates with increasing canopy height. In the first process, each species might have used more substrates in more complex vegetation. This within‐species plasticity would then lead to higher individual‐based behavioral functional diversity in sites with higher canopy. We tested this hypothesis only in the two species with most foraging records, the Brown Thornbill and the Yellow‐faced Honeyeater, but it was not supported in either of them. In the second process, foraging substrates might have been added as canopy increased in a regular, nested pattern. With within‐species plasticity of substrate use excluded, this would mean that species with specific substrate use strategies were added nonrandomly in more complex habitats. Indeed, our analyses of nestedness and turnover were consistent with this hypothesis. However, to fully evaluate this possibility, we would need to know species‐specific patterns of fine foraging substrate use in all the 111 species we recorded across sites. Such data are currently not available. However, the combination of a regular, nested increase in the number of foraging substrates used with comparably high turnover of species is remarkable. It leads us to believe that the accumulation of additional species with increasing canopy height must have been nonrandom in one way or another. If that was the case, we witnessed a remarkable case of convergent accumulation of ecological functions with vegetation complexity on the background of substantial, continent‐wide biogeographic turnover of species.

## CONFLICT OF INTEREST

The authors declare no conflict of interest.

## AUTHOR CONTRIBUTIONS

**Vladimír Remeš:** Conceptualization (lead); Formal analysis (lead); Funding acquisition (lead); Investigation (equal); Methodology (lead); Project administration (lead); Writing‐original draft (lead); Writing‐review & editing (lead). **Eva Remešová:** Data curation (lead); Investigation (equal); Writing‐review & editing (supporting). **Nicholas R. Friedman:** Investigation (equal); Writing‐review & editing (supporting). **Beata Matysioková:** Investigation (equal); Writing‐review & editing (supporting). **Lucia Rubáčová:** Investigation (equal); Writing‐review & editing (supporting).

## Supporting information

Supplementary Material

## Data Availability

All data can be accessed on Zenodo under https://doi.org/10.5281/zenodo.4588389.
