# Supplementary material for: Functional diversity of avian communities increases with canopy height: From individual behavior to continental‐scale patterns
Source: Ecol Evol. 2021 Jul 27;11(17):11839–51. doi: 10.1002/ece3.7952 (PMC8427649; doi:10.1002/ece3.7952)
Supplement: Supplementary file 1 — Supplementary Material [file ECE3-11-11839-s001.pdf]

Supporting information

Functional diversity of avian communities increases with canopy height: from individual behavior to continental-scale patterns

Vladimír Remeš, Eva Remešová, Nicholas R. Friedman, Beata Matysioková, and Lucia Rubáčová

Appendix S1 Detailed description of field methods

Appendix S2 Comparison of functional diversity indices

Appendix S3 Combinations of foraging behaviors for calculation of functional diversity

Appendix S4 Geographic effects (latitude and distance)

Appendix S5 Statistical results of path analyses

Appendix S6 Analyses of rarefied estimates of functional diversity

Appendix S7 Distance-based analyses

Appendix S8 Within-species analyses of substrate use diversity

Appendix S9 Analyses of nestedness and turnover

Table S1 Overview of our 21 sites.

Figure S1 Map of eastern Australia with our 21 sites

Figure S2 Vegetation profiles of our 21 sites

Figure S3 Summary vegetation characteristics of our 21 sites

Figure S4 Results of the PCA analysis applied on the vegetation characteristics

Appendix S1 Detailed description of field methods

2.1 Study areas and vegetation sampling

We worked at 21 sites during the austral breeding season from September to December in 2016 and 2017 (Table S1). Similar seasonal timing means that our sampling was standardised in terms of seasonal effects, but also that we cannot take seasonal differences in foraging behaviour into account (e.g. Recher and Davis 2014, Recher 2016). All sites were located in eucalypt woodlands and open-forests of eastern Australia (Johnson 2006; Fig 1; for the names of the sites see Fig. S1). We thus limited the habitat breadth that we investigated by avoiding i) shrubby and grassland vegetation in more arid parts of Australia, and ii) rainforests due to their thick vegetation, which precludes easy observation of foraging birds and thus requires much more intensive sampling than was possible in our study. We selected habitats with native vegetation, within protected areas (mostly National Parks), and without any agricultural activity including grazing by livestock (Table S1). At each site, we delimited three transects. Each transect was 2 km long and 50 m wide (10 ha) and was divided into two 25m wide sides – right and left – that were divided by the path or narrow forest road along which we walked. We placed the transects such that they were representative of local vegetation and had homogeneous vegetation cover, with three spatial replicates at each site. Transects were usually at least 1 km apart. In general, it was not easy to find sites and transects with desired characteristics and thus our site selection was influenced by convenience (availability of sites, access to sites, availability of paths or small roads). In spite of these problems, we achieved a large-scale coverage of eucalypt woodlands and open forests across eastern Australia (Figs. 1 and S1).

In order to quantify vegetation structure at each site, we used a systematic sampling design by placing 10 points (200 m apart) along each transect. At each point, we delimited a semi-circle with the radius of 25 m (area ca. 0.1 ha or 1000 m^2) with alternating left and right direction from the transect. Thus, the total area surveyed for vegetation structure was ca. 3 ha (i.e., 30000 m^2) on each site (3 transects x 10 points x 1000 m^2). At each point, we recorded vegetation cover in five *height strata* (0–1 m, 1.1–2.0 m, 2.1–5 m, 5.1–10 m, >10 m) and vegetation cover and height of four *vegetation strata* (herbaceous, shrub, subcanopy, and canopy). Height strata were delimited by a priori selected height bands, while vegetation strata were determined by major vertical vegetation layers typical of woodlands and forests. Thus, for vegetation strata, besides cover we also needed to measure the height of individual strata. The height of the herbaceous stratum was estimated visually, while the height of shrub, subcanopy, and canopy strata was measured by taking three measurements by a laser rangefinder (Nikon Forestry). Vegetation cover of each band (height strata) or stratum (vegetation strata) was estimated by eye on the scale ranging from 0 (no vegetation) to 10

(fully covered). The estimate was done independently by two observers who then converged on a consensus estimate.

2. 2 Foraging behaviour

Each transect was walked twice by two observers with 0–3 days between the two sessions. The two observers worked on different sides of the transect and swapped sides between the two sessions. We started observations shortly after dawn and walked along the transect with roughly constant speed for 4 h, thus standardizing observation effort. We limited observations to days without rain and strong wind. We recorded foraging behaviour of all passerines (Passeriformes) with the exception of individuals foraging high above the canopy ("aerial foragers"), which were mostly swallows and martins (Hirundinidae) and woodswallows (Artamidae), although woodswallows also forage on vegetation and the ground. We constantly and systematically scanned all vegetation for birds. We thus aimed to minimize bias introduced by locating only singing or otherwise conspicuous individuals. We located most of the birds by sight without using auditory cues (n = 1959), while 188 birds were detected due to singing and 470 due to vocalizing (usually contact voices among members of a group). Once we located a bird, we counted to five before recording its behaviour to avoid bias towards recording conspicuous behaviours. If it did not forage within 1 min, we left it and continued searching for another bird. We were interested in patterns of resource partitioning, and thus did not record the process of searching for food, but only an event of actually procuring or attempting to procure food (*prey attack*). First records of prey attacks might sometimes differ from subsequent ones (Recher and Gebski 1990) and thus sampling more prey attacks from the same individual could be useful. On the other hand, this could lead to underestimating uncertainties in quantifying foraging behaviour (Hejl et al. 1990). We thus compromised and for each individual recorded at most three prey attacks (mean = 2.25 attacks per individual bird, n = 2624 individual birds). For each prey attack, we recorded bird species (or genus, if species identification was impossible), foraging method and substrate, foraging height, height of the plant the bird foraged on, distance from the plant stem, and foliage density around the foraging bird.

In terms of behaviour, we recognized eight types of *foraging methods* used by birds for attacking the prey that we adapted from previous studies of foraging in Australian birds (e.g. Recher et al. 1985, Ford et al. 1986):

1. *Gleaning* – moving on/through the substrate and taking prey from its surface; prey is taken while the bird is on the substrate (e.g. many thornbills, Acanthizidae and honeyeaters, Meliphagidae)
2. *Hang-gleaning* – gleaning while the bird is hanging upside-down (e.g. Striated Thornbill, *Acanthiza lineata* and Silvereye, *Zosterops lateralis*)

3. *Snatching* – moving on/through the substrate and making short flights to take the prey from nearby substrates; prey is taken while the bird is in the air (e.g. Rufous Whistler, *Pachycephala rufiventris*)
4. *Hover-snatching* – snatching while the bird stays in the air (hovers) when taking the prey from a substrate (e.g. Weebill, *Smicrornis brevirostris*)
5. *Probing* – extracting food from/within thick or deep substrate, such as soil, litter or flowers (e.g. flower-feeding honeyeaters)
6. *Manipulation* – includes variety of methods such as scratching, digging and tearing to expose the prey (e.g. Crested Shrike-Ttit, *Falcunculus frontatus* and Australo-Papuan babblers, Pomatostomidae)
7. *Pouncing* – direct flight from a perch to the site where the prey is taken (usually ground), whereby the bird lands and takes the prey; it may continue flying afterwards (e.g. butcherbirds, Cracticidae or Australasian robins, Petroicidae)
8. *Flycatching* (same as Hawking or Sallying) – flying from a substrate to take a flying prey, whereby both the foraging bird and prey are in the air (e.g. monarch flycatchers, Monarchidae or fantails, Rhipiduridae)

We recognized eight main categories and 17 fine categories (i.e., subcategories) of foraging *substrate* from which the birds collected food (subcategories are in parentheses):

1. *Ground* (three subcategories: *bare ground*, *leaf litter*, *grass*)
2. *Leaf* (two subcategories: *small leaves* – any dimension below 10 cm, *large leaves*)
3. *Bark* (four subcategories: *twig* – bears leaves at the end of a branch, *small branch* – diameter below 10 cm, large branch, *trunk* – vertical stem supporting a shrub or a tree)
4. *Bud* (unopened leaf or flower; no subcategories)
5. *Flower* (any size or type; no subcategories)
6. *Fruit* (including dry and fleshy fruits; no subcategories)
7. *Air* (four subcategories: *above trees* – but not high in the sky, *between trees* – between canopies of shrubs and trees, *within trees* – within a canopy, *over ground* – below 1 m high)
8. *Other* (includes special substrates such as spider webs; no subcategories).

Plant height and *foraging height* of each prey attack was measured by a laser rangefinder (Nikon Forestry). When we recorded more than one prey attack of the same individual, we estimated the average height of all of them. When the bird foraged on a shrub or a tree, we distinguished the following four categories of *distance from stem* – directly on the stem, inner half of the shrub/tree crown, outer half of the crown, and on the outer edge of the crown. Finally, we recognized three

categories of the *foliage density* around the foraging bird – low (including zero), medium (bird still easy to see) and high (bird difficult to see).

References in Appendix S1

Ford H.A., Noske S., Bridges L. (1986) Foraging of birds in eucalypt woodland in north-eastern New South Wales. Emu 86:168–179.

Hejl S.J., Verner J., Bell, G.W. (1990) Sequential versus initial observations in studies of avian foraging. Studies in Avian Biology 13:166–173.

Johnson B. (2006) Woodlands and open-forests of Eastern Australia. In 'Ecology: An Australian Perspective.' 2nd edn. (Eds P. Attiwill and B. Wilson.) pp. 515–534. (Oxford University Press: Sydney.)

Recher H.F. (2016) The winter foraging behaviour of birds in a mixed eucalypt forest and woodland of the Southern Tablelands of New South Wales. Australian Zoologist 38:1–15.

Recher H.F., Davis W.E. Jr. (2014) Response of birds to episodic summer rainfall in the Great Western Woodlands, Western Australia. Australian Zoologist 37:206–224.

Recher H.F., Gebski V. (1990) Analysis of the foraging ecology of eucalypt forest birds: Sequential versus single-point observations. Studies in Avian Biology 13:174–180.

Recher H.F., Holmes R.T., Schulz M., Shields J., Kavanagh, R. (1985) Foraging patterns of breeding birds in eucalypt forest and woodland of southeastern Australia. Australian Journal of Ecology 10:399–419.

Appendix S2 Comparison of functional diversity indices

We calculated functional richness (IN 1.2. in Table 1 of Schleuter et al. 2010), functional evenness (IN 2.1), functional divergence (IN 3.3), and niche breadth indices of Levins, Simpson, and Shannon (Krebs 1999). For functional richness, we also calculated its value using only "frequently used" resources, where the cutoff is set usually as (1/no of categories), but never >10% (Schleuter et al. 2010). These indices are designated as "FRXperc" in the following figures, where X = the cutoff point in percents (e.g., "FR5perc" means cutoff at 5%). All these indices correlated positively and thus we chose only the Shannon index for all subsequent analyses of functional diversity. The reasons were twofold. First, Shannon index correlated most closely with all other indices (Figs. S2-1 to S2-9 below). Second, we used rarefaction to account for unequal sampling across sites for both species richness and functional diversity, and rarefaction is available only for Shannon’s and Simpson’s index (Hsieh et al. 2016).

For explanations of the categories of behavior we used to calculated functional diversity, see Appendix S3.

References in Appendix S2

Hsieh T.C., Ma K.H., Chao, A. (2016) iNEXT: an R package for rarefaction and extrapolation of species diversity (Hill numbers). Methods in Ecology and Evolution, 7:1451-1456.

Krebs, C. (1999) Ecological methodology, 2ed. Benjamin Cummings.

Schleuter D., Daufresne M., Massol F., Argillier C. (2010) A user's guide to functional diversity indices. Ecological Monographs, 80:469-484.

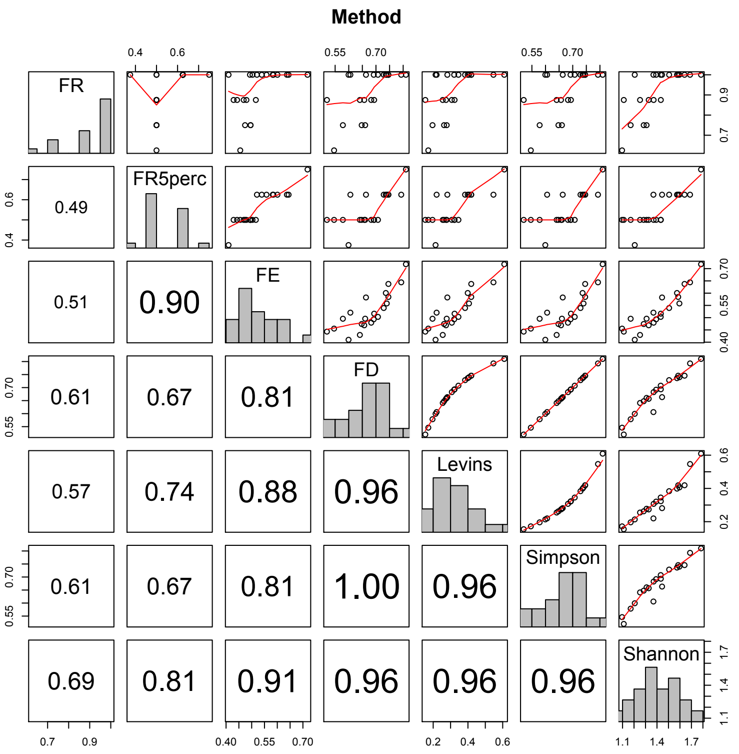

Figure S2-1 Correlations of functional diversity indices calculated for our 21 study sites using foraging method (n = 8).

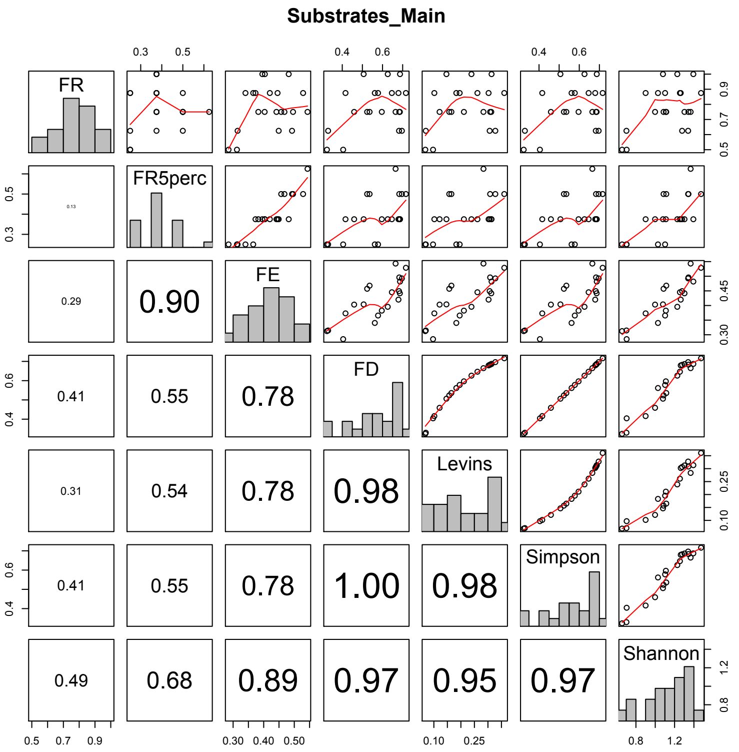

Figure S2-2 Correlations of functional diversity indices calculated for our 21 study sites using main foraging substrates (n = 8).

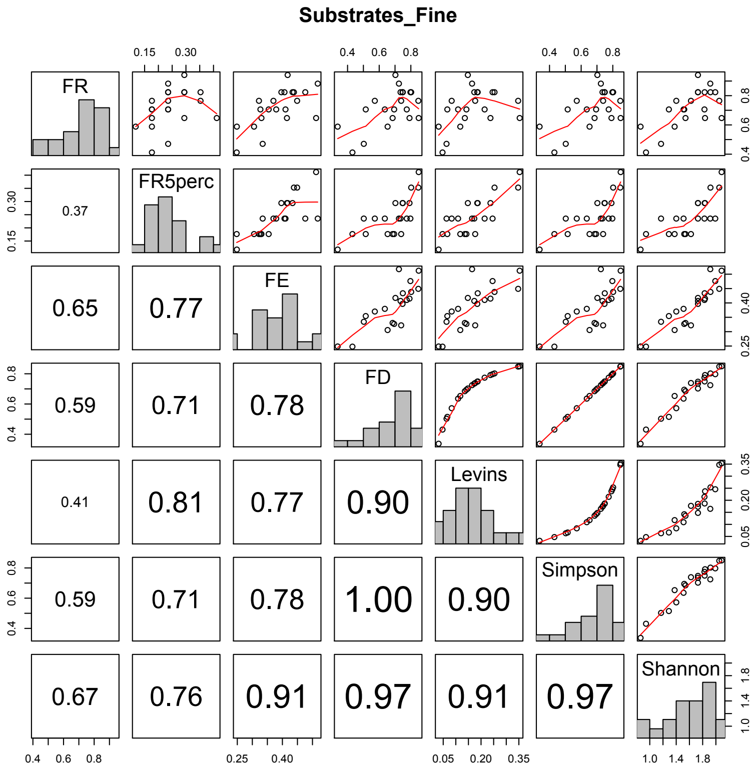

Figure S2-3 Correlations of functional diversity indices calculated for our 21 study sites using detailed foraging substrates (n = 17).

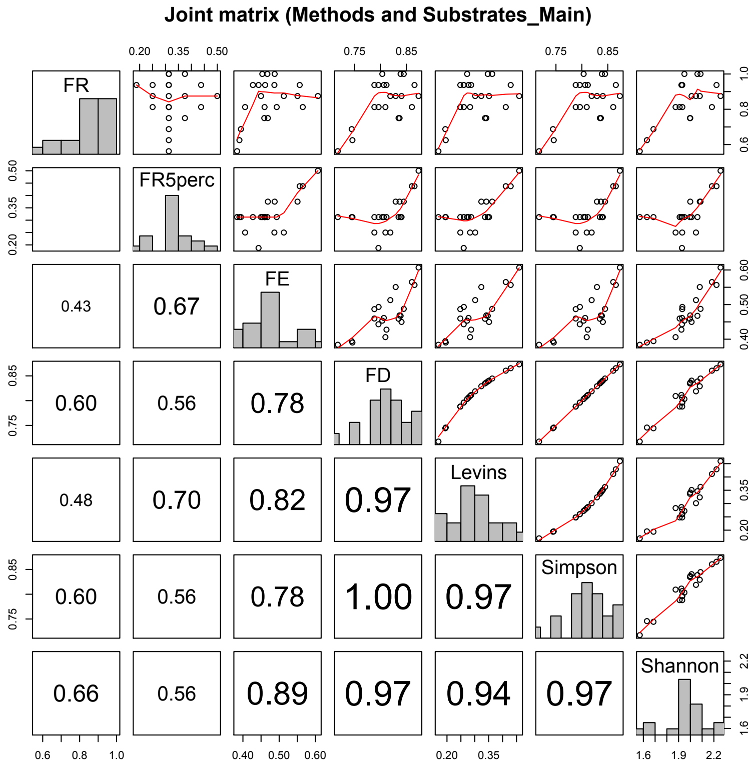

Figure S2-4 Correlations of functional diversity indices calculated for our 21 study sites using foraging methods and main substrates together in a joint matrix (n = 16).

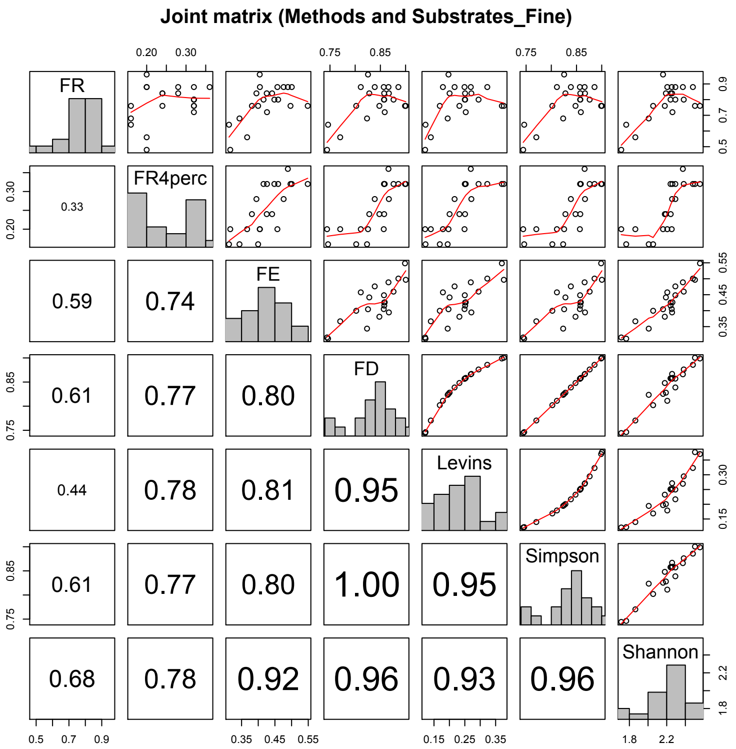

Figure S2-5 Correlations of functional diversity indices calculated for our 21 study sites using foraging methods and detailed substrates together in a joint matrix (n = 25).

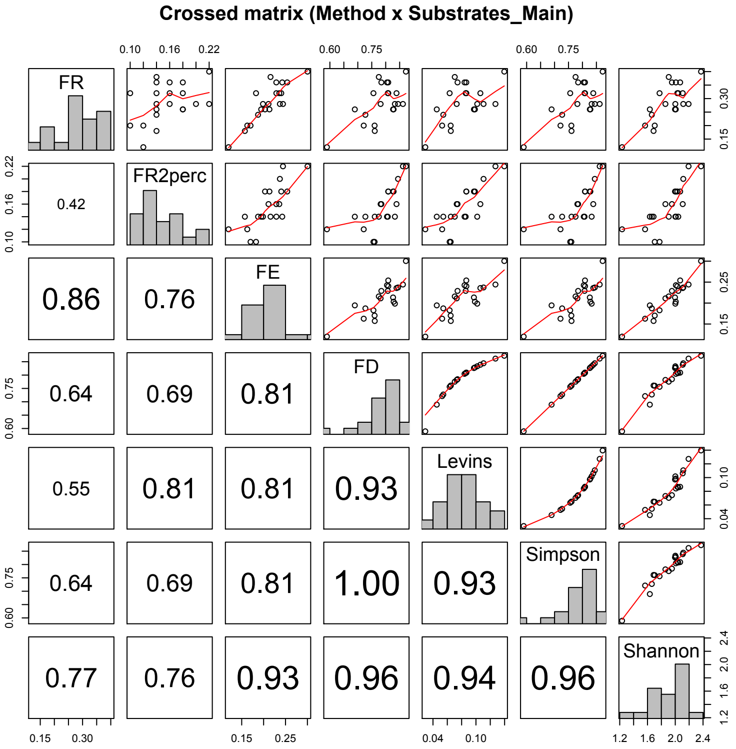

Figure S2-6 Correlations of functional diversity indices calculated for our 21 study sites using foraging methods and main substrates in a crossed matrix (n = 50).

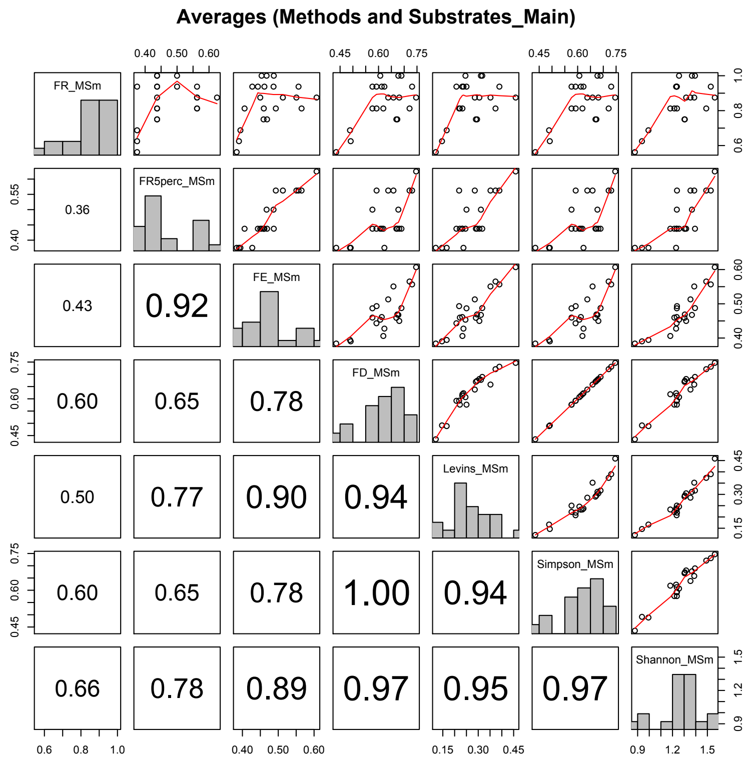

Figure S2-7 Correlations of functional diversity indices calculated for our 21 study sites using foraging methods and main substrates separately, then averaged (n = 8 and 8).

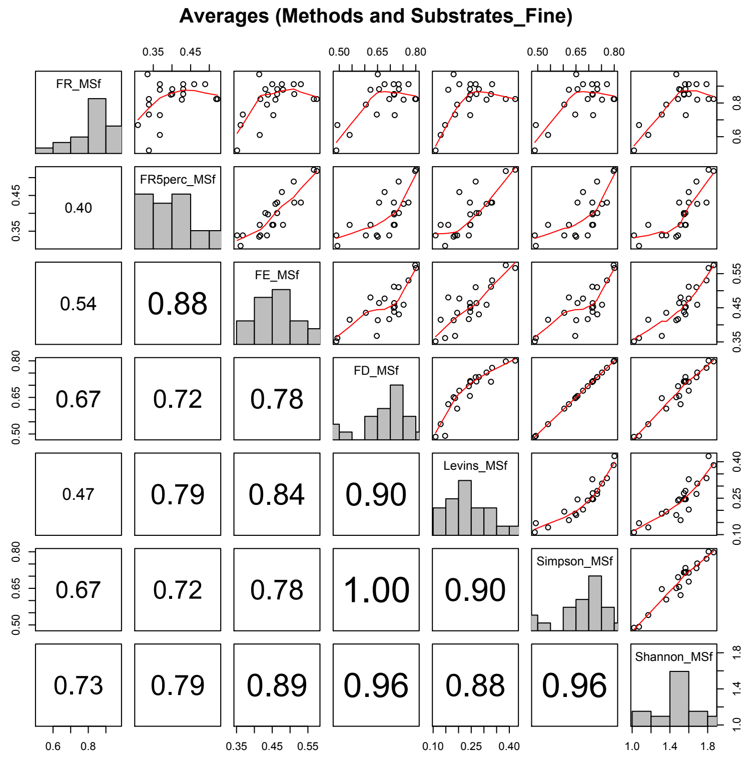

Figure S2-8 Correlations of functional diversity indices calculated for our 21 study sites using foraging methods and detailed substrates separately, then averaged (n = 8 and 17).

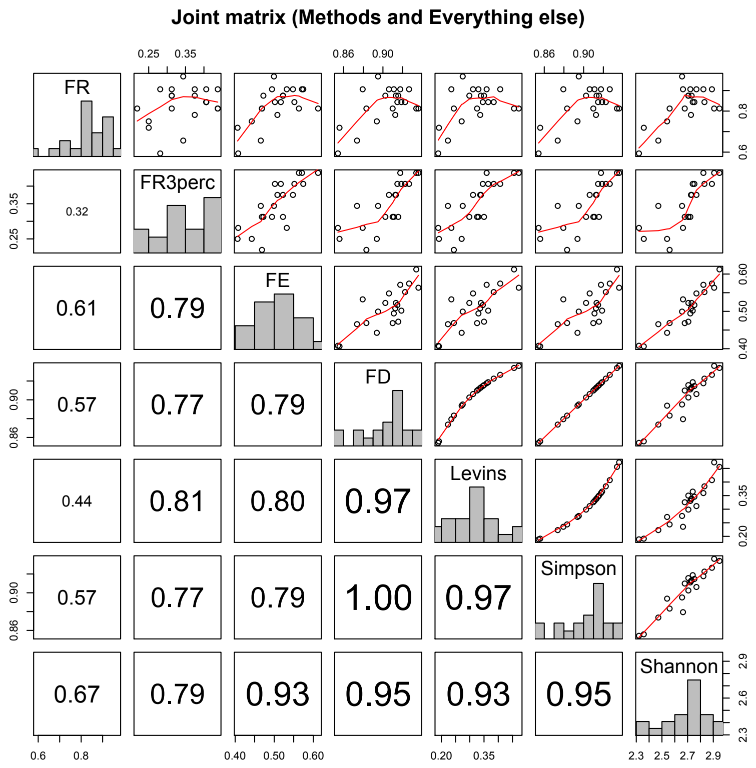

Figure S2-9 Correlations of functional diversity indices calculated for our 21 study sites using foraging methods, detailed substrates, distance from stem, and foliage density in a joint matrix (n = 32).

Appendix S3 Combinations of foraging behaviors for calculation of functional diversity (FD)

We defined several combinations of behavioral traits we recorded in the field. These were:

1) Foraging method (acronym “Method” in figures below; n = 8)
2) Main foraging substrate (“SubMain”; n = 8)
3) Joint matrix of method and main foraging substrate (“Method_SubMain”; n = 16)
4) Separate matrices of methods and main substrates (“Avg_Method_SubMain”; n = 8 and 8)
5) Crossed matrix of methods and main substrates (“Method_x_SubMain”; n = 50)
6) Detailed foraging substrate (“SubFine”; n = 17)
7) Joint matrix of method and detailed foraging substrate (“Method_SubFine”; n = 25)
8) Separate matrices of methods and detailed substrates (“Avg_Method_SubFine”; n = 8 and 17)
9) Joint matrix of foraging methods, detailed substrates, distance from stem, and foliage density (“Everything”; n = 32)

Joint matrix means that traits were concatenated into a single matrix for all study sites and FD indices were calculated using this matrix. *Crossed matrix* means that we calculated how many foraging records per site there were in a particular combination of each foraging method and substrate (of which there were 50 plausible ones; combinations implausible by definition include e.g. “flycatching on the ground”). These combinations were treated as new behavioral categories and FD indices were calculated using these new behavioral categories. This approach is uniquely possible only with foraging records obtained in the field (see Supplement S3 in Harmáčková et al. 2019). *Separate matrices* means that FD indices were calculated separately using the two respective matrices and then averaged per study site.

We show correlations of FD indices across different combinations of behavioral traits. These are functional richness (Fig. S3-1), functional evenness (Fig. S3-2), functional divergence (Fig. S3-3), and Levin’s (Fig. S3-4) and Shannon diversity indices of niche breadth (Fig. S3-5). We do not show any figure for Simpson’s index, because its value is numerically identical to functional divergence (see Figures S2-1 to S2-9 in Appendix S2).

References in Appendix S3

Harmáčková L., Remešová E., Remeš V. (2019) Specialization and niche overlap across spatial scales: Revealing ecological factors shaping species richness and coexistence in Australian songbirds. Journal of Animal Ecology 88:1766-1776.

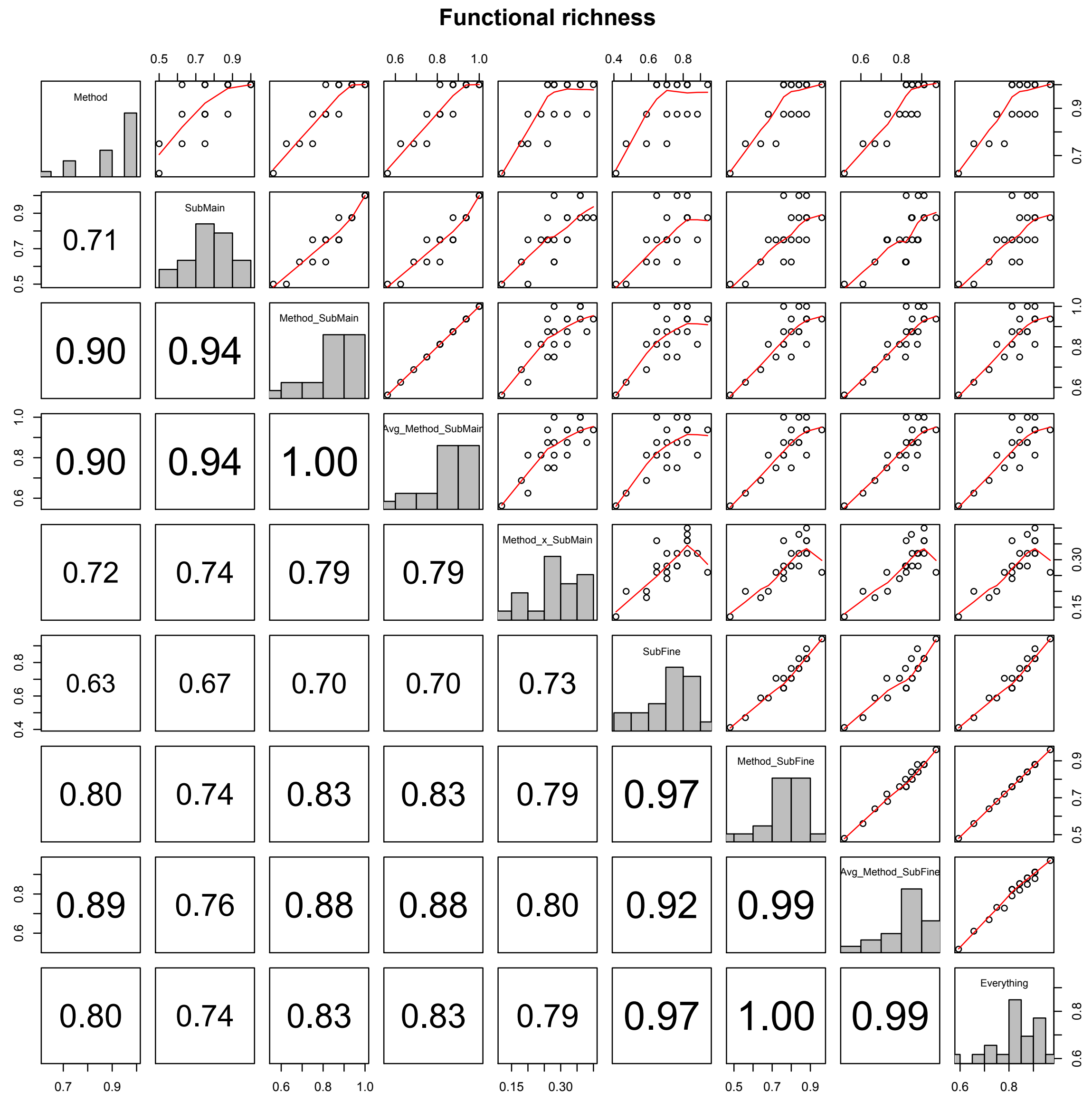

Figure S3-1 Correlations of functional richness calculated for our 21 study sites for different combinations of behavioral traits.

Functional evenness

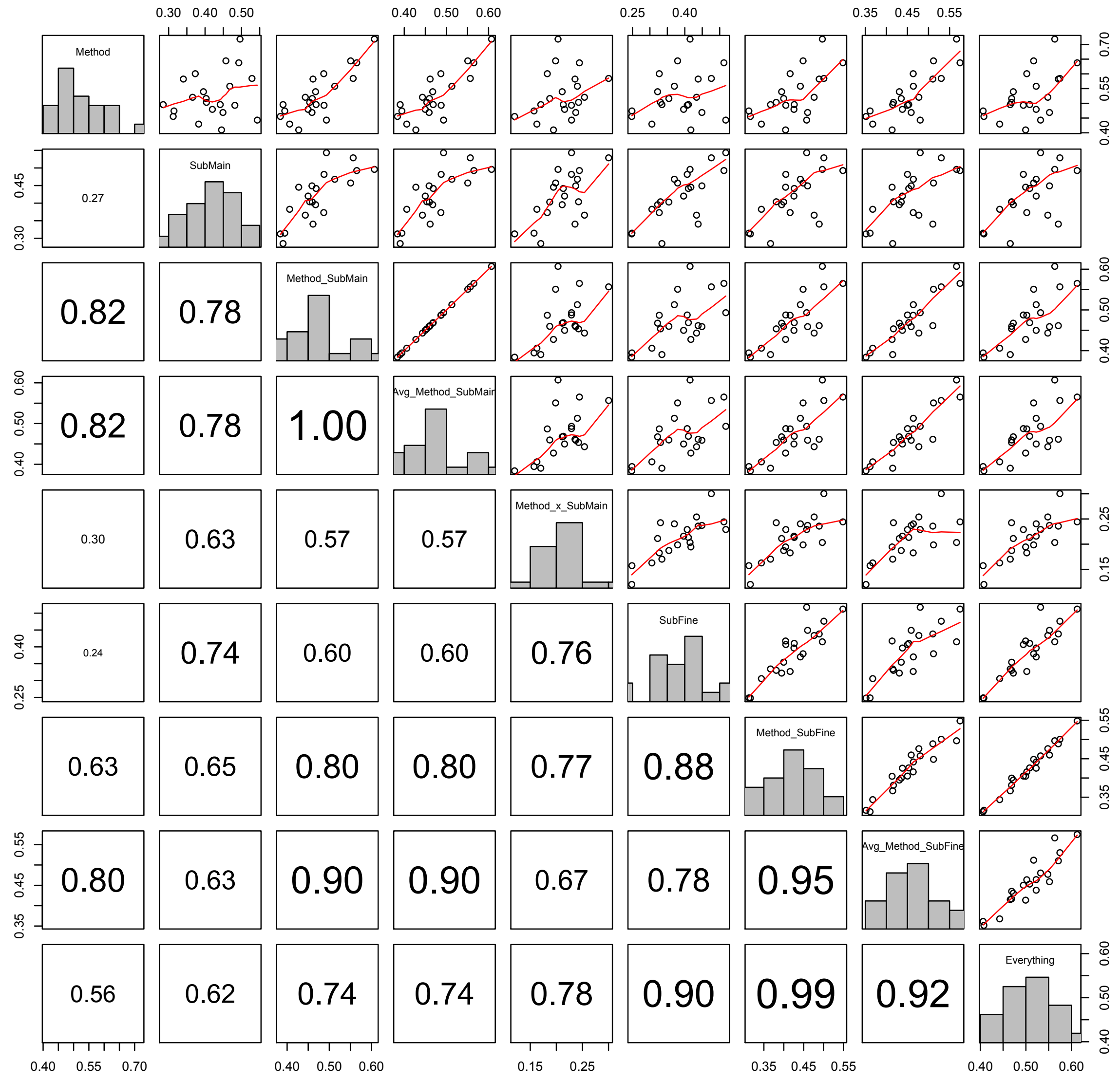

Figure S3-2 Correlations of functional evenness calculated for our 21 study sites for different combinations of behavioral traits.

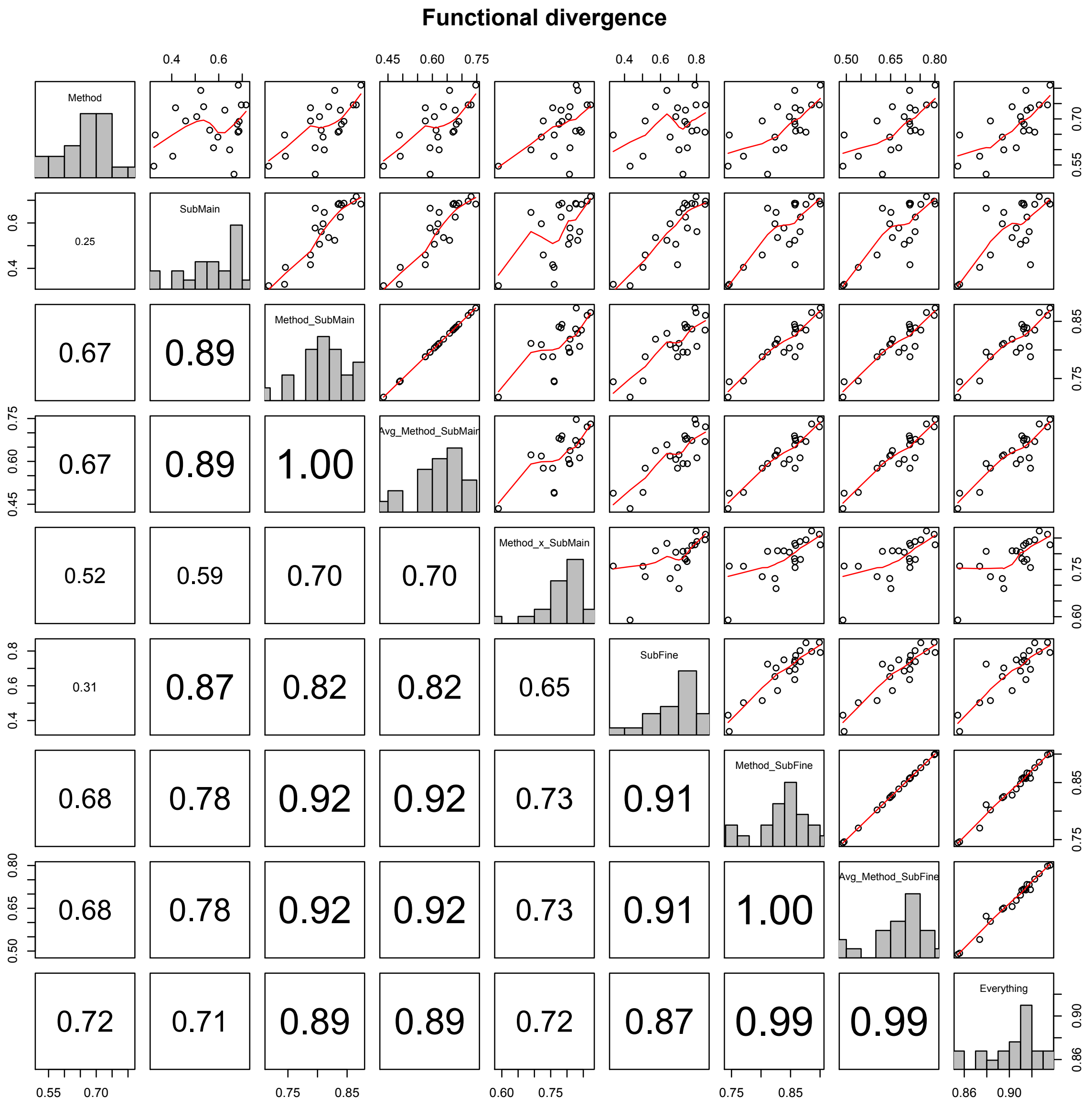

Figure S3-3 Correlations of functional divergence calculated for our 21 study sites for different combinations of behavioral traits.

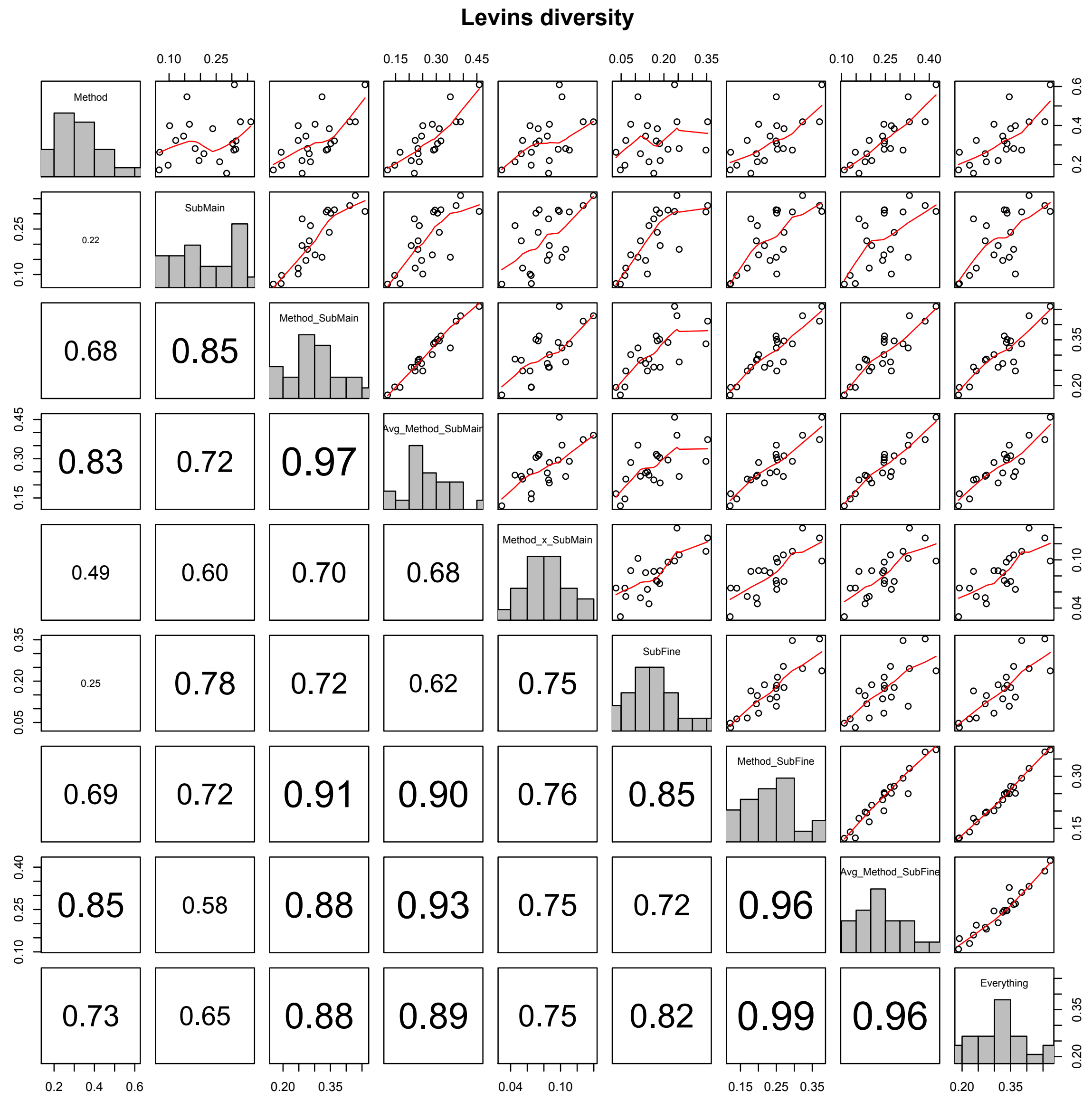

Figure S3-4 Correlations of the Levins' index of niche breadth calculated for our 21 study sites for different combinations of behavioral traits.

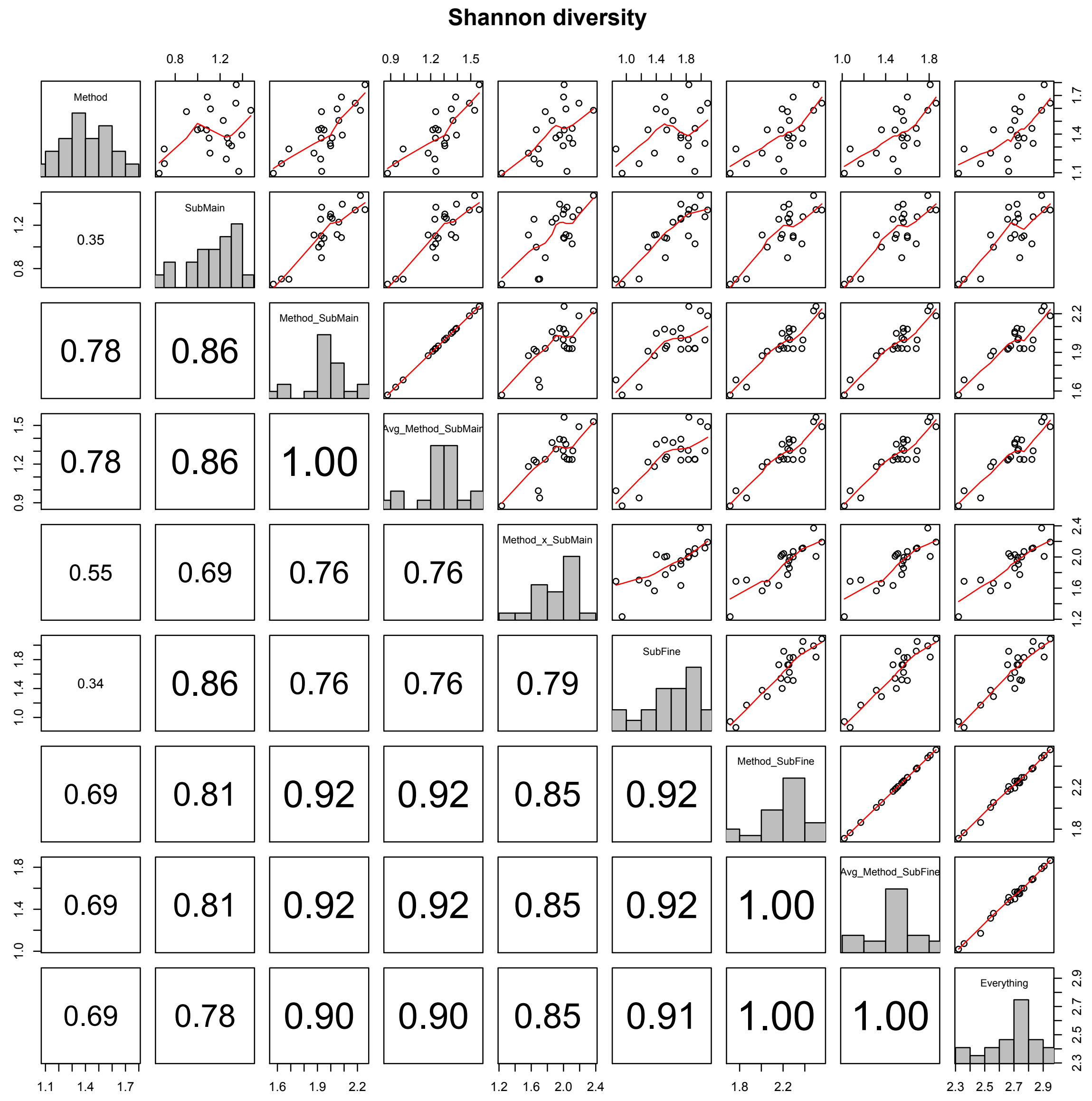

Figure S3-5 Correlations of the Shannon's index of niche breadth calculated for our 21 study sites for different combinations of behavioral traits.

Appendix S4 Geographic effects (latitude and distance)

Latitude

Australia has a peculiar east-west climatic gradient instead of the usual north-south gradient. Accordingly, there was no correlation between vegetation structure and latitude (log10(canopy height) vs latitude $r = -0.27$, $p = 0.25$; PC1 of vegetation structure vs latitude $r = -0.13$, $p = 0.59$). Similarly, there is virtually no latitudinal gradient in species richness in eastern Australia (e.g. Remeš and Harmáčková 2018). Accordingly, there was no correlation between species richness and latitude in our data ($r = 0.15$, $p = 0.52$). The same was true for total density ($r = -0.15$, $p = 0.51$), Shannon behavioral diversity (foraging methods: $r = -0.11$, $p = 0.63$; substrates: $r = -0.24$, $p = 0.30$; method-substrate combinations: $r = -0.21$, $p = 0.36$), and morphological hypervolume-based disparity ($r = 0.32$, $p = 0.16$). Thus, since latitude correlated neither with our predictors nor with our dependent variables, it could not confound our analyses.

Geographic distance

There are many metrics that are available to quantify pairwise multivariate distances between sites, with appropriate distance metrics selected based on the type of data. We calculated distance matrices using:

i) Euclidean distances for continuous measurements (vegetation characteristics)

ii) Bray-Curtis distances for categorical traits (foraging behavior)

iii) Shortest geographical distance in km (geographical location) – these were the same irrespective of the package we used; we finally used the *fields* package (Nychka et al. 2017).

iv) Total beta diversity for species abundances. There are many indices of pairwise beta diversity calculated on species abundances across sites. We used packages *betapart* (function "beta.pair.abund"; Baselga et al. 2018), *BAT* (function "beta"; Cardoso et al. 2018), and *vegan* (function "vegdist"; Oksanen et al. 2018) to calculate several beta diversity indices and compare them. The reasons were twofold. First, we wanted to make sure that most beta diversity indices gave the same information. Second, there is considerable debate about the best beta diversity measures in the literature. For example, Cardoso et al. (2015) explicitly say that they do not agree with the framework proposed by Baselga and Orme (2012). However, there seems to be considerable agreement across the approaches proposed to calculate total beta diversity on species abundances (Fig. A4-1; the same is true for the nestedness vs. turnover/richness component of the total beta diversity, not shown). We picked the Sorensen index calculated in the *BAT* package for further analyses.

Pairwise distances were organized into distance matrices. We analyzed them using multiple regressions on distance matrices in the *ecodist* package (Goslee and Urban 2007).

References in Appendix S4

Baselga A., Orme, C.D.L. (2012) betapart: an R package for the study of beta diversity. Methods in Ecology and Evolution 3:808–812.

Baselga A., Orme D., Villeger S., De Bortoli J., Leprieur F. (2018) betapart: Partitioning Beta Diversity into Turnover and Nestedness Components. R package version 1.5.1. https://CRAN.R-project.org/package=betapart

Cardoso P., Rigal F., Carvalho J.C. (2015) BAT–Biodiversity Assessment Tools, an R package for the measurement and estimation of alpha and beta taxon, phylogenetic and functional diversity. Methods in Ecology and Evolution, 6:232-236.

Cardoso P., Rigal F., Carvalho J.C. (2018) BAT: Biodiversity Assessment Tools. R package version 1.6.0. https://CRAN.R-project.org/package=BAT

Goslee S.C., Urban D.L. 2007. The ecodist package for dissimilarity-based analysis of ecological data. Journal of Statistical Software 22(7):1-19.

Nychka D., Furrer R., Paige J., Sain S. (2017) “fields: Tools for spatial data.” doi: 10.5065/D6W957CT (URL: http://doi.org/10.5065/D6W957CT), R package version 9.6, <URL: www.image.ucar.edu/~nychka/Fields>.

Oksanen J., Blanchet F.G., Friendly M., Kindt R., Legendre P., McGlinn D., Minchin P.R., O'Hara R.B., Simpson G.L., Solymos P., Stevens M.H.H., Szoecs E., Wagner H. (2018) vegan: Community Ecology Package. R package version 2.5-3. https://CRAN.R-project.org/package=vegan

Remeš V., Harmáčková L. (2018) Disentangling direct and indirect effects of water availability, vegetation, and topography on avian diversity. Scientific Reports 8:15475.

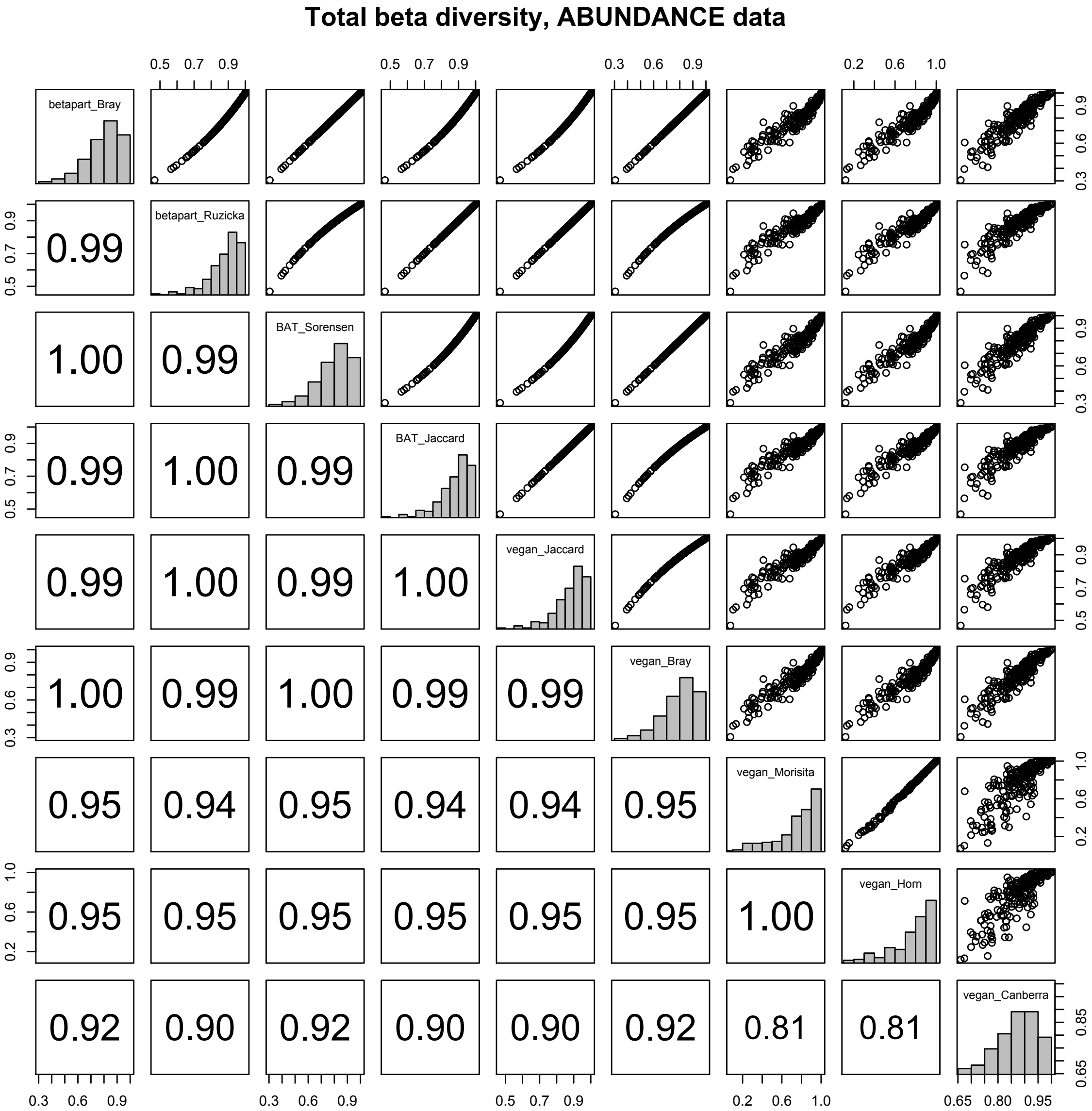

Figure S4-1 Comparison of different pairwise indices of total beta diversity calculated on species abundances across all 21 study sites. The first part of the name along the diagonal denotes the R package, while the second part denotes the beta diversity index.

Appendix S5 Statistical results of path analyses

We tested the following linear equations within a Structural Equation Model (SEM):

1) Species richness ~ log10(Canopy height) + Total density,

2) Total density ~ log10(Canopy height),

3) Functional diversity ~ log10(Canopy height) + Species richness + Total density,

where Functional diversity was expressed as the Shannon diversity index.

Test results for the first two equations were the same within all SEMs (R^2 = 0.53 for SR and 0.18 for Density)

Response	Predictor	Estimate	Std.Error	DF	Crit.Value	P.Value
SR	logCanopy	0.4027	0.1794	21	2.2452	0.038
SR	Density	0.4560	0.1794	21	2.5422	0.020
Density	logCanopy	0.4294	0.2072	21	2.0728	0.052

The following tables present results for the third equation, estimated for morphology and behavioral traits.

Results for morphological disparity ("Morphology"; R^2 = 0.83)

Response	Predictor	Estimate	Std.Error	DF	Crit.Value	P.Value
Morphology	logCanopy	-0.0483	0.1270	21	-0.3806	0.708
Morphology	SR	1.0502	0.1475	21	7.1211	<0.001
Morphology	Density	-0.2037	0.1308	21	-1.5574	0.138

Results for behavioral diversity of foraging methods ("Method"; R^2 = 0.20):

Response	Predictor	Estimate	Std.Error	DF	Crit.Value	P.Value
Method	logCanopy	0.0695	0.2714	21	0.2562	0.801
Method	SR	0.3850	0.3153	21	1.2211	0.239
Method	Density	0.0296	0.2797	21	0.1057	0.917

Results for behavioral diversity of method-substrate combinations ("Met x Sub"; R^2 = 0.50)

Response	Predictor	Estimate	Std.Error	DF	Crit.Value	P.Value
Met x Sub	logCanopy	0.3524	0.2149	21	1.6394	0.120
Met x Sub	SR	0.3925	0.2496	21	1.5724	0.134
Met x Sub	Density	0.0649	0.2215	21	0.2929	0.773

Results for behavioral diversity of main foraging substrates ("Sub_Main"; R^2 = 0.44)

Response	Predictor	Estimate	Std.Error	DF	Crit.Value	P.Value
Sub_Main	logCanopy	0.0286	0.2267	21	0.1260	0.901
Sub_Main	SR	0.3456	0.2633	21	1.3126	0.207
Sub_Main	Density	0.3739	0.2335	21	1.6011	0.128

Results for behavioral diversity of fine foraging substrates ("Sub_Fine"; R^2 = 0.44)

Response	Predictor	Estimate	Std.Error	DF	Crit.Value	P.Value
Sub_Fine	logCanopy	0.4038	0.2279	21	1.7722	0.094
Sub_Fine	SR	0.0334	0.2647	21	0.1262	0.901
Sub_Fine	Density	0.3488	0.2348	21	1.4856	0.156

Results for behavioral diversity of all foraging substrates (“Sub_All”; R^2 = 0.47)

Response	Predictor	Estimate	Std.Error	DF	Crit.Value	P.Value
Sub_All	logCanopy	0.4513	0.2219	21	2.0339	0.058
Sub_All	SR	-0.0213	0.2577	21	-0.0828	0.935
Sub_All	Density	0.3734	0.2286	21	1.6331	0.1208

Acronyms:

Estimate	Standardized effect size
Std.Error	Standard error of the estimate
SR	Species richness
Density	Total density of all individuals
logCanopy	log10(Canopy height)

Appendix S6 Analyses of rarefied estimates of functional diversity

We calculated the expected species richness and behavioral diversity for a standardized total density using individual-based rarefaction. Total density varied from 49 to 220 individuals across sites. The maximum recommended extrapolation extent is to twice the number of individuals. We thus standardized species richness to 100 individuals. The number of foraging records varied from 112 to 522 (because we recorded up to three foraging events per individual, see above). We standardized behavioral diversity to 225 individuals. Rarefaction was not available for the morphological hypervolume-based disparity. Estimates (i.e., effect sizes) are standardized for comparison.

Foraging methods (R^2 adj = 0.05)

Predictor	Estimate	Std. Error	t value	Pr(>|t|)
Canopy height	0.1151	0.2573	0.447	0.660
Species richness	0.3100	0.2573	1.205	0.244

Foraging method-main substrates combinations (R^2 adj = 0.36)

Predictor	Estimate	Std. Error	t value	Pr(>|t|)
Canopy height	0.3860	0.2113	1.827	0.0843
Species richness	0.3587	0.2113	1.698	0.1067

Main foraging substrates (R^2 adj = 0.20)

Predictor	Estimate	Std. Error	t value	Pr(>|t|)
Canopy height	0.1858	0.2372	0.783	0.444
Species richness	0.4019	0.2372	1.695	0.107

Fine foraging substrates (R^2 adj = 0.31)

Predictor	Estimate	Std. Error	t value	Pr(>|t|)
Canopy height	0.5407	0.2199	2.459	0.024
Species richness	0.1218	0.2199	0.554	0.586

All foraging substrates (R^2 adj = 0.32)

Predictor	Estimate	Std. Error	t value	Pr(>|t|)
Canopy height	0.5870	0.2180	2.693	0.015
Species richness	0.0630	0.2180	0.289	0.776

Appendix S7 Distance-based analyses

We calculated distance matrices using (see Appendix S4 for justifications):

i) Euclidean distances for continuous measurements (vegetation characteristics)

ii) Bray-Curtis distances for categorical traits (foraging behavior)

iii) Shortest geographical distance in km (geographical location)

iv) Total beta diversity for species abundances using the Sorensen index

Estimates of pairwise correlation coefficients between the distance matrices are apparent from Fig. S7-1. We tested these relationships using multivariate tests on matrices in the *ecodist* (Goslee and Urban 2007) and *phytools* packages (Revell 2012). *Ecodist* implements the multiple regression on distance matrices (MRM) approach (Lichstein 2007), while *phytools* uses multiple matrix regression using the partial Mantel test. These approaches gave identical results, so we report only the results using the “MRM” (*ecodist*) function.

Results of the MRM test on multiple distance matrices:

	Forag. Methods (R^2 = 0.19)		Forag x Substr (R^2 = 0.18)		Substrates_Main (R^2 = 0.13)	
Predictor	Correlation	P-val	Correlation	p-val	Correlation	p-val
Vegetation	0.039	0.021	0.032	0.046	0.003	0.843
Beta_diversity	0.173	0.016	0.199	0.010	0.219	0.006
Geography	-0.0001	0.404	-0.0001	0.522	-0.0001	0.184

“Met_Sub” denotes method-main substrate combinations.

	Substrates_Fine (R^2 = 0.30)		Substrates_All (R^2 = 0.31)	
Predictor	Correlation	P-val	Correlation	p-val
Vegetation	0.042	0.018	0.028	0.016
Beta_diversity	0.307	0.001	0.209	0.002
Geography	-0.0001	0.209	-0.0001	0.188

References in Appendix S7

Goslee S.C., Urban D.L. 2007. The ecodist package for dissimilarity-based analysis of ecological data. Journal of Statistical Software 22(7):1-19.

Lichstein J. 2007. Multiple regression on distance matrices: A multivariate spatial analysis tool. Plant Ecology 188: 117-131.

Revell L. J. (2012) phytools: An R package for phylogenetic comparative biology (and other things). Methods Ecol. Evol. 3 217-223.

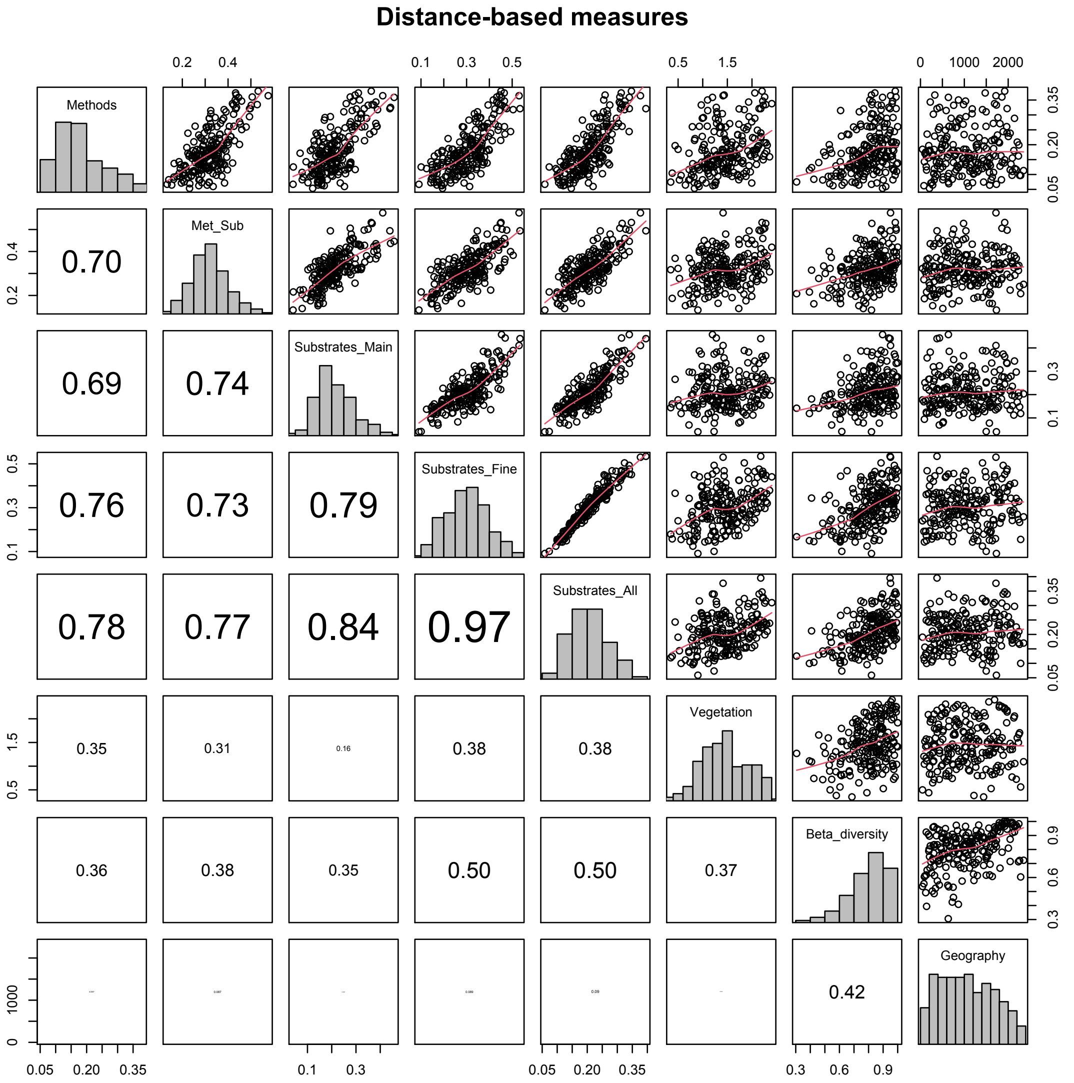

Figure S7-1 Pairwise correlations of distances between pairs of our 21 study sites. Distance-based measures are based on foraging behavior, vegetation structure, species composition (Beta_diversity), and geography (distance in km). “Met_Sub” denotes foraging method-main substrate combinations.

Appendix S8 Within-species analyses of substrate use diversity

Substrate use diversity increased with increasing canopy height across study sites. One possible explanation is that each species might use more substrates in more complex vegetation. We thus tested the within-species relationship between the diversity in substrate use and canopy height, controlled for the number of foraging records per species per site. We did this only for the Brown Thornbill (*Acanthiza pusilla*, n = 263 foraging records) and the Yellow-faced Honeyeater (*Caligavis chrysops*, n = 193), the two species with the highest number of foraging records available (Fig. A8-1).

Brown Thornbills were observed at 13 sites and used 11 out of 17 categories of fine substrates, while Yellow-faced Honeyeaters were observed on 16 sites and used 10 categories of fine substrate. For the Brown Thornbill, Shannon diversity index of fine substrate use increased with the number of foraging records (standardized effect size, ES = 0.67, $p = 0.017$), while the relationship with canopy height was not significant (ES = 0.01, $p = 0.979$). For the Yellow-faced Honeyeater, neither canopy height (ES < 0.01, $p = 0.987$) nor the number of foraging records (ES = 0.20, $p = 0.473$) predicted significantly Shannon diversity in fine substrate use.

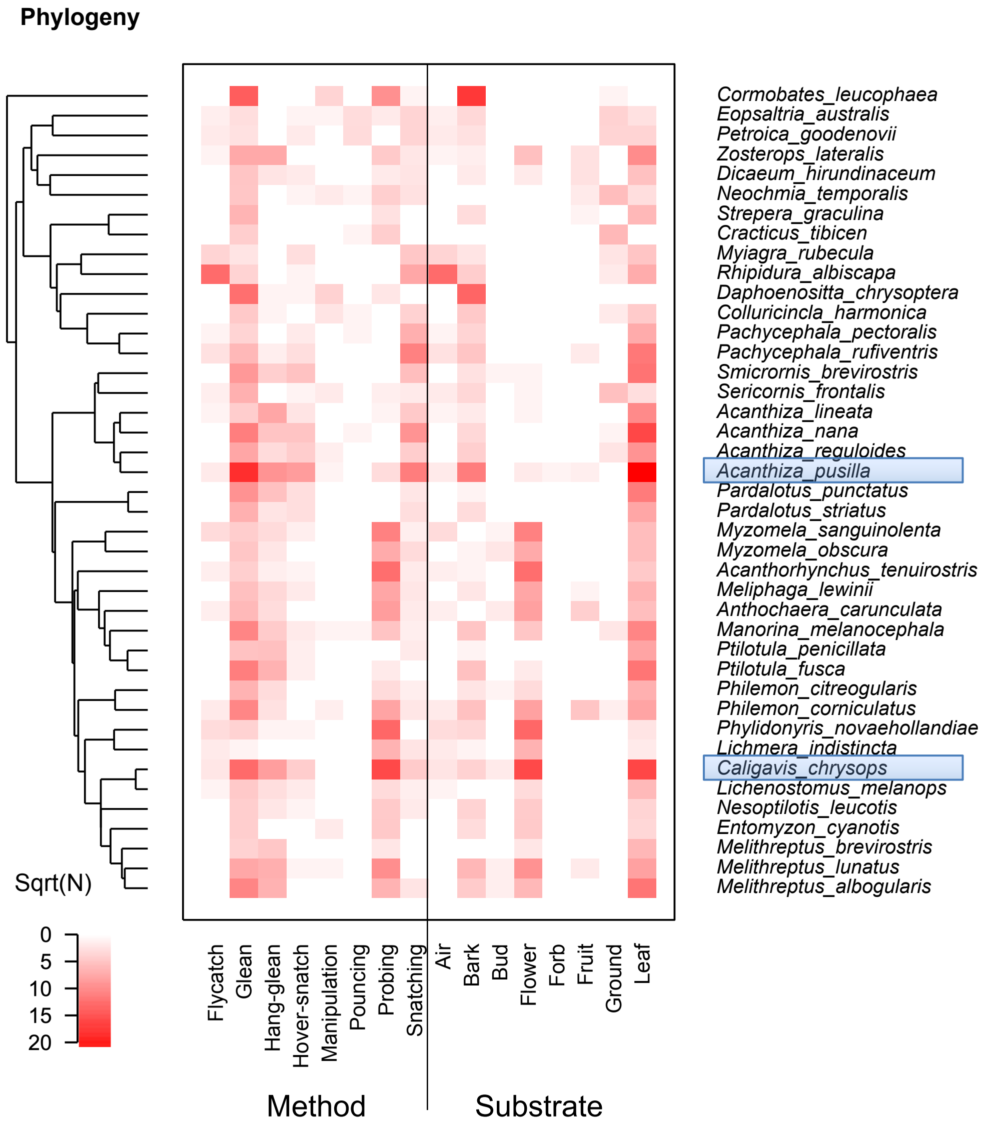

Figure S8-1 Foraging methods and main substrates used by 41 species with at least 30 foraging records available. Species are mapped on a phylogeny. Two species in which we analyzed within-species relationship between diversity in fine substrate use and vegetation complexity are highlighted by blue rectangles (Brown Thornbill, *Acanthiza pusilla*, and Yellow-faced Honeyeater, *Caligavis chrysops*).

Appendix S9 Analyses of nestedness and turnover

The number of substrates used increased with increasing canopy height across study sites (Fig. S9-1). Certain substrates were used in (almost) all sites and in high numbers, e.g. small and large leaves, and flowers. Others were used predominantly in sites with high canopy, e.g. litter and trunk. Only a few substrates were added in sites with low canopy, e.g. buds. This pattern is consistent with high nestedness and low turnover of substrate use (Fig. S9-1).

Nestedness and turnover components of total beta diversity of fine substrate use (mean (SD)).

	BAT package		*betapart* package	
Type of data	Nestedness	Turnover	Nestedness	Turnover
Presence-absence	0.59 (0.35)	0.41 (0.35)	0.57 (0.37)	0.43 (0.37)
Abundance	0.52 (0.30)	0.48 (0.30)	0.43 (0.31)	0.57 (0.31)

The number of species also increased with increasing canopy height across study sites (Figs. S9-2 and S9-3). However, there was not a set of species that would be present across all sites, due to biogeographic turnover of avifaunas. There is a set of species present at all sites with canopy height above ca. 20 m, but otherwise the pattern has more turnover than nestedness compared to foraging substrates. An interesting feature is the occurrence of specific sets of species on only certain sites, e.g. Paluma or Herberton Rg. (Fig. S9-3).

Nestedness and turnover components of total beta diversity of species composition (mean (SD)).

	BAT package		*betapart* package	
Type of data	Nestedness	Turnover	Nestedness	Turnover
Presence-absence	0.25 (0.17)	0.75 (0.17)	0.11 (0.11)	0.89 (0.11)
Abundance	0.26 (0.18)	0.74 (0.18)	0.07 (0.09)	0.93 (0.09)

Partitioning of substrate use and species composition into turnover and nestedness components was calculated in the *betapart* (Baselga et al. 2018) and *BAT* (Cardoso et al. 2018) packages. As the authors of these packages do not agree on the best partitioning algorithm, we present results of both approaches. We used both presence-absence and abundance data.

References in Appendix S9

Baselga A., Orme D., Villeger S., De Bortoli J., Leprieur F. (2018) betapart: Partitioning Beta Diversity into Turnover and Nestedness Components. R package version 1.5.1. https://CRAN.R-project.org/package=betapart

Cardoso P., Rigal F., Carvalho J.C. (2018) BAT: Biodiversity Assessment Tools. R package version 1.6.0. https://CRAN.R-project.org/package=BAT

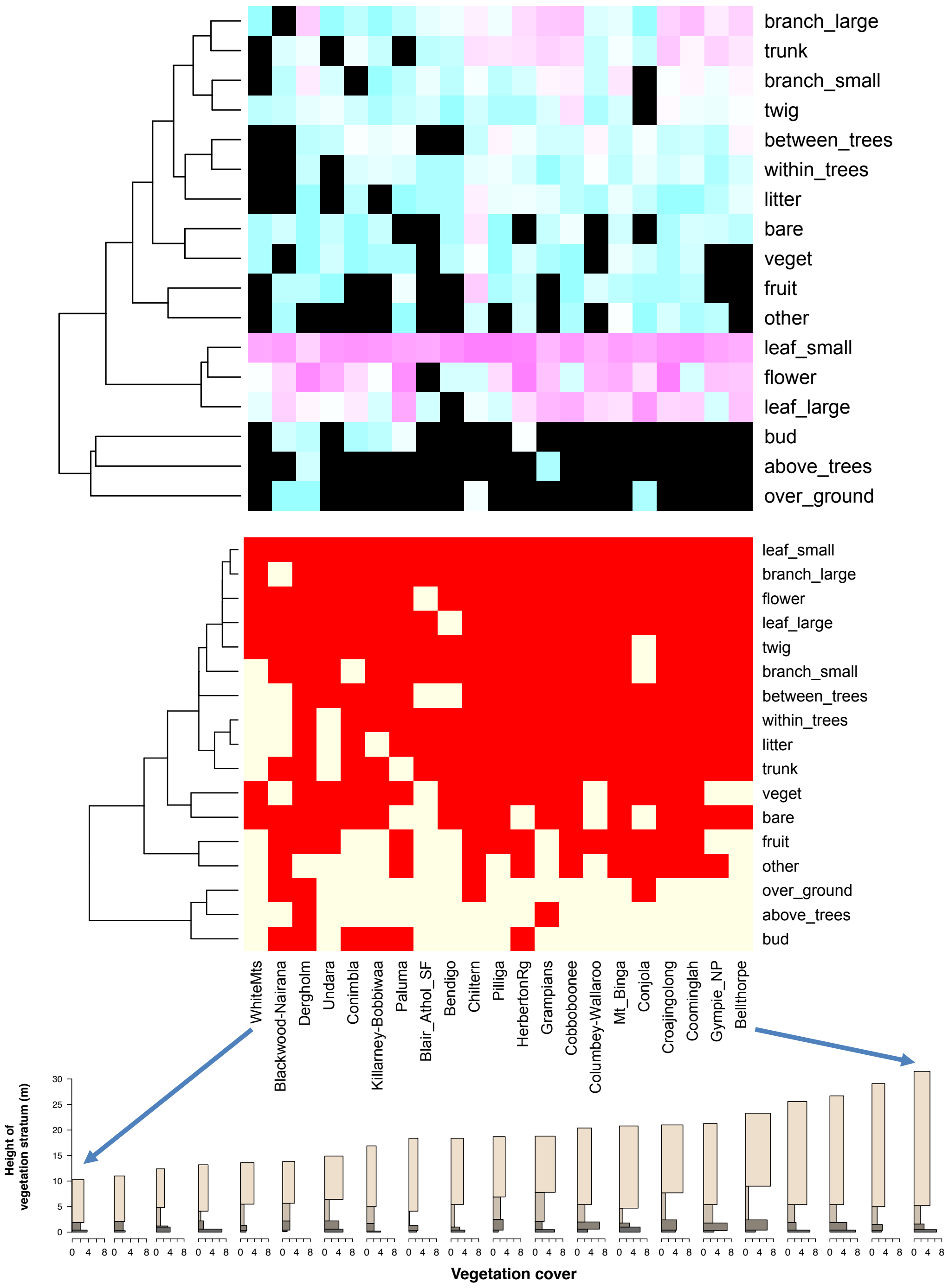

Fig. S9-1 "Abundance" (top) and occurrence (bottom) of substrate use across our 21 study sites. Abundance is coded from absent (black) to few records (dark blue) to many records (dark violet). Occurrence is codes as present (red) vs. absent (yellow). Sites are ordered from the lowest (left) to the highest canopy (right; vegetation profiles at the bottom). Fine foraging substrates along the vertical axes (n = 17 categories) are clustered based on similarity of their abundance/occurrence across sites.

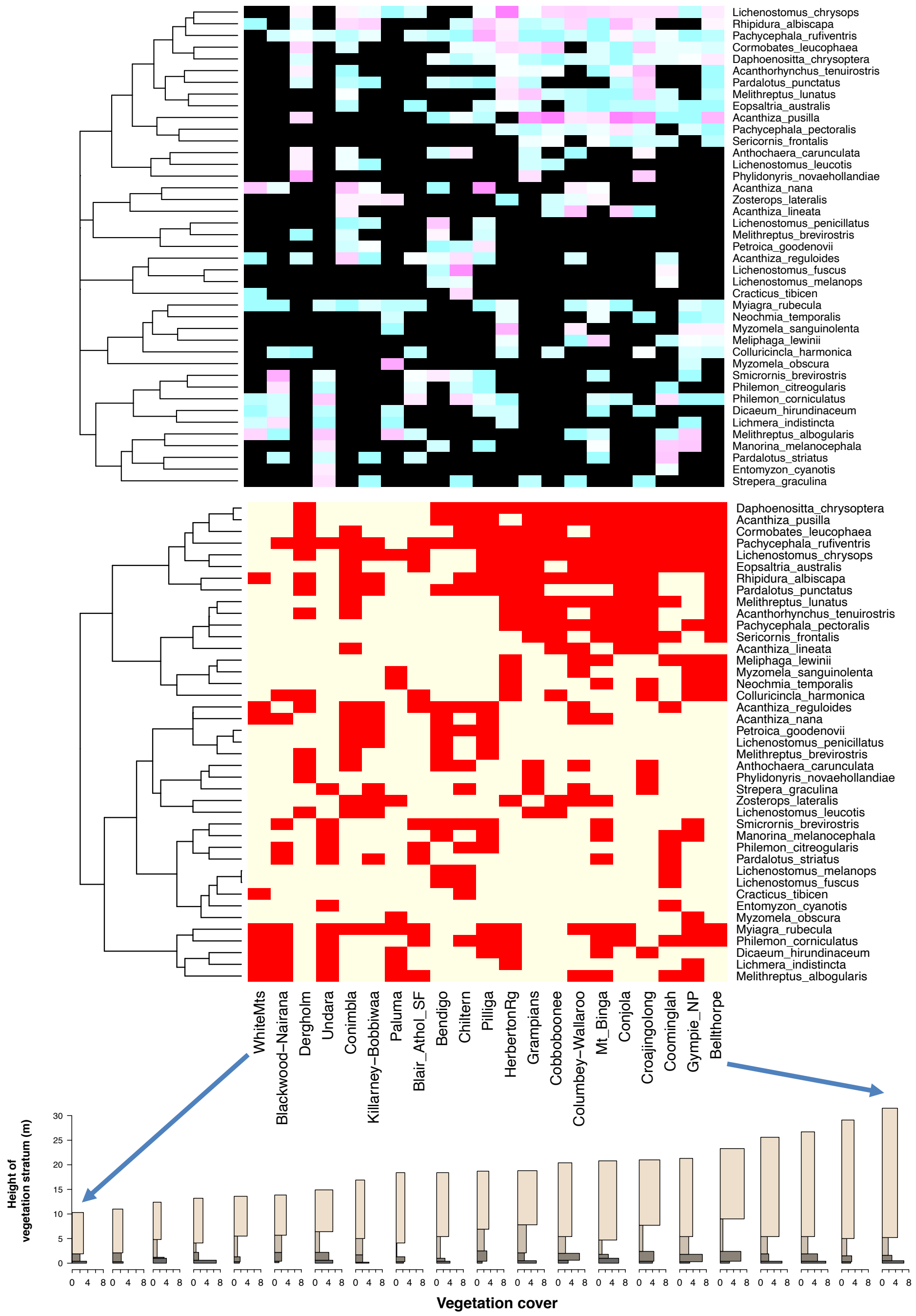

Fig. S9-2 Abundance (top) and occurrence (bottom) of the 41 species with at least 30 foraging records across our 21 study sites. Abundance is coded from absent (black) to few records (dark blue) to many records (dark violet). Occurrence is codes as present (red) vs. absent (yellow). Sites are ordered from the lowest (left) to the highest canopy (right; vegetation profiles at the bottom). Species are clustered based on similarity of their abundance/occurrence across sites.

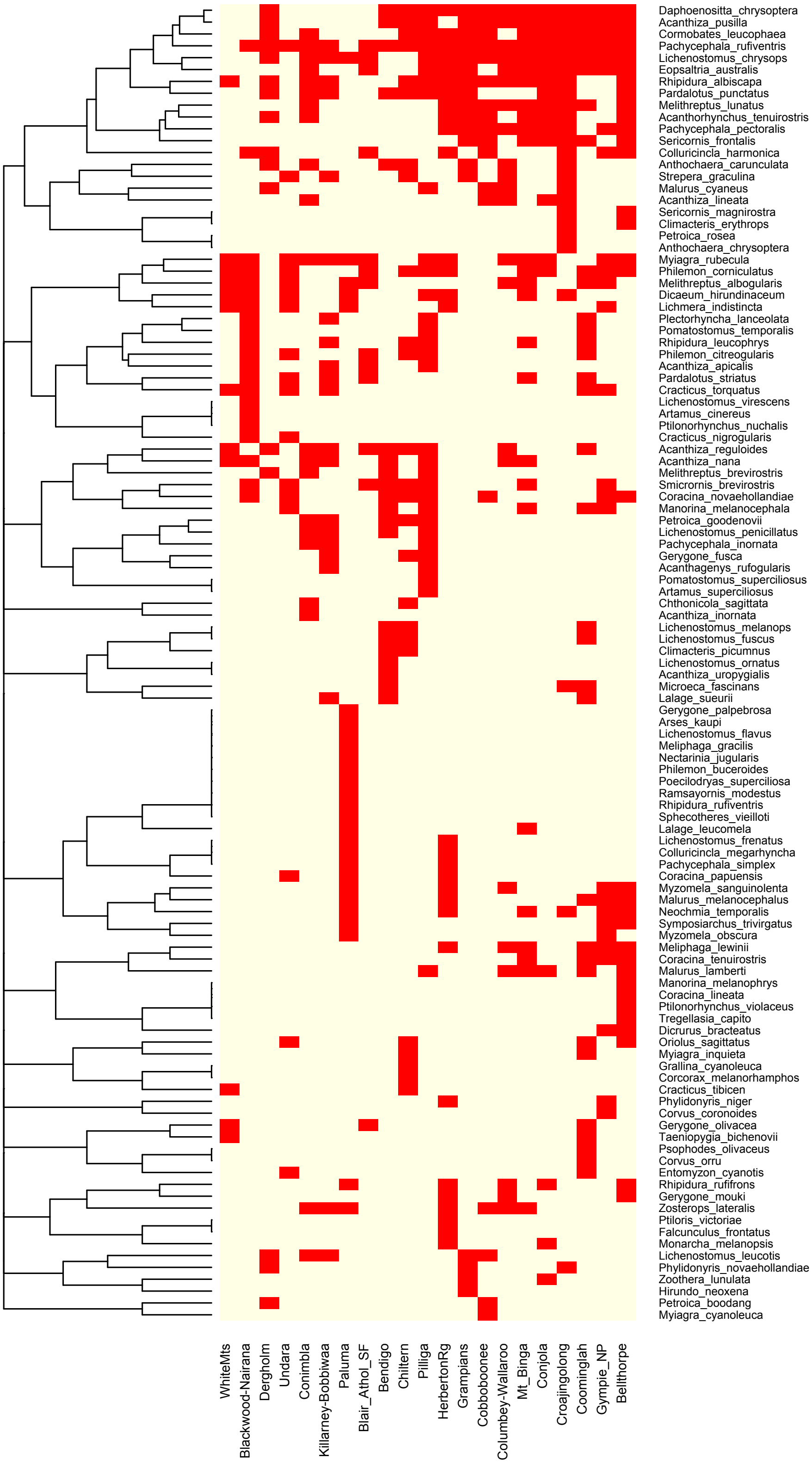

Figure S9-3 Occurrence of all 111 species recorded across our 21 study sites. Occurrence is codes as present (red) vs. absent (yellow). Sites are ordered from the lowest (left) to the highest canopy (right). Species are clustered based on similarity of their occurrence across sites.

Table S1 Overview of the sites, year and month(s) of visit, state, geographic longitude and latitude, and observers (ER: E. Remešová, VR: V. Remeš, BM: B. Matysioková, LR: L. Rubáčová). Sites are ordered by canopy height (from lowest to highest) as in Figures S1 and S2.

Site	Year	Months	State	Latitude	Longitude	Observers
White Mountains NP	2016	IX - X	QLD	-20.714	145.177	ER, VR
Blackwood NP/ Nairana NP	2016	X	QLD	-21.465	146.683	ER, VR
Dergholm SP	2016	XI	VIC	-37.254	141.140	BM, LR
Undara Volcanic NP	2016	IX	QLD	-18.208	144.572	ER, VR
Conimbla NP	2017	X	NSW	-33.894	148.391	ER, VR
Killarney NP/ Bobbiwaa SCA	2017	IX	NSW	-30.240	149.884	ER, VR
Paluma Range NP	2016	X	QLD	-18.982	146.244	ER, VR
Blair Athol SF	2017	XII	QLD	-22.697	147.474	BM, LR
Bendigo SF	2016	XI	VIC	-36.704	144.352	BM, LR
Chiltern-Mt. Pilot NP	2016	X	VIC	-36.137	146.577	BM, LR
Pilliga East NP	2017	IX - X	NSW	-30.546	149.595	ER, VR
Herberton Range NP	2016	X - XI	QLD	-17.333	145.415	ER, VR
Grampians NP	2016	XI	VIC	-37.225	142.554	BM, LR
Cobboboonee NP	2016	XI	VIC	-38.151	141.514	BM, LR
Columbey NP/ Wallaroo NP	2017	X	NSW	-32.581	151.741	ER, VR
Mt. Binga NP	2017	XI	QLD	-27.001	151.915	BM, LR
Conjola NP	2017	X - XI	NSW	-35.165	150.429	ER, VR
Croajingolong NP	2016	XII	VIC	-37.518	149.697	BM, LR
Coominglah SF	2017	XI	QLD	-24.897	151.040	BM, LR
Gympie NP	2017	X	QLD	-26.054	152.704	BM, LR
Bellthorpe NP	2017	XI	QLD	-26.877	152.696	BM, LR

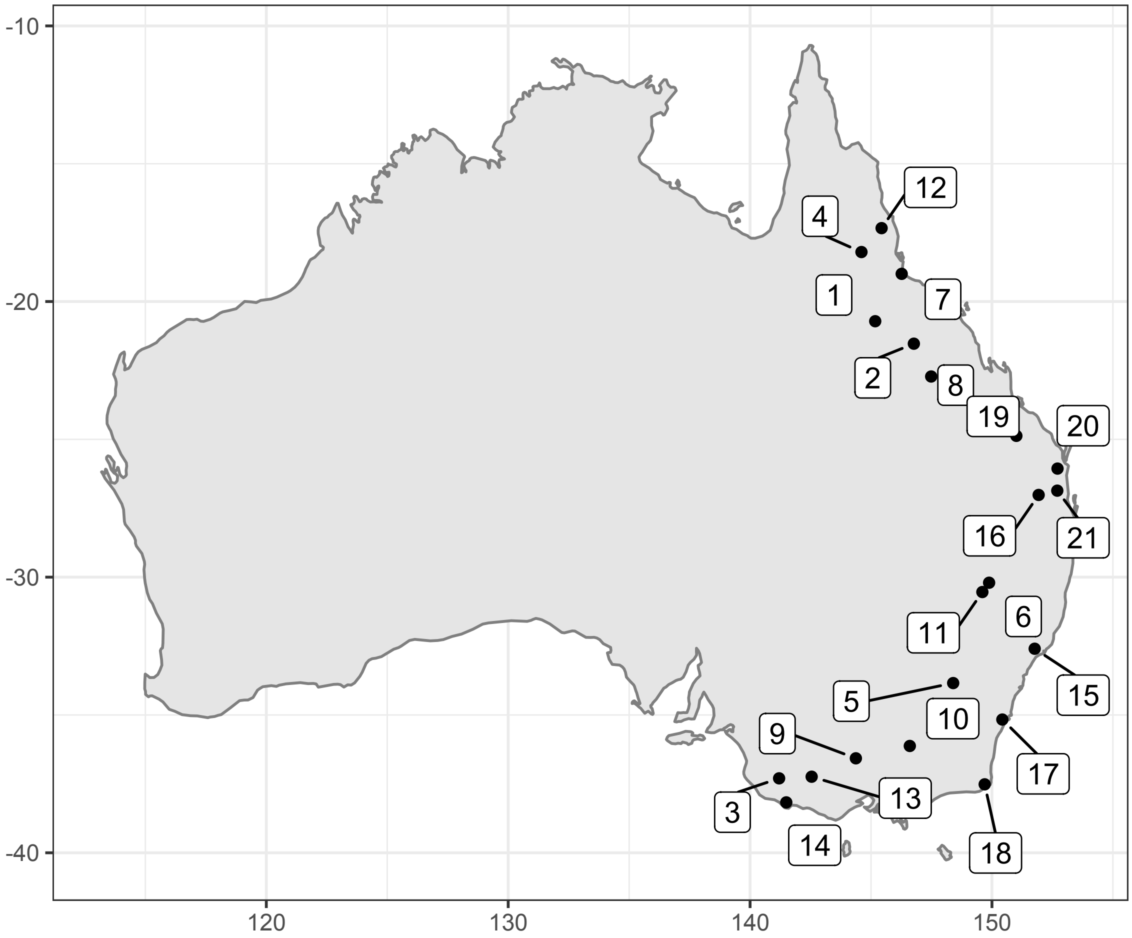

Figure S1 Map of Australia with our 21 study sites. Size of the circle is proportional to the number of foraging records while colour refers to the mean canopy height at the site. The number in brackets encodes the site name as follows: (1) White Mountains NP, (2) Blackwood NP/ Nairana NP, (3) Dergholm SP, (4) Undara Volcanic NP, (5) Conimbla NP, (6) Killarney NP/ Bobbiwaa SCA, (7) Paluma Range NP, (8) Blair Athol SF, (9) Bendigo SF, (10) Chiltern-Mt. Pilot NP, (11) Pilliga East NP, (12) Herberton Range NP, (13) Grampians NP, (14) Cobboboonee NP, (15) Columbey NP/ Wallaroo NP, (16) Mt. Binga NP, (17) Conjola NP, (18) Croajingolong NP, (19) Coominglah SF, (20) Gympie NP, (21) Bellthorpe NP. Sites are numbered with increasing canopy height as in Table S1.

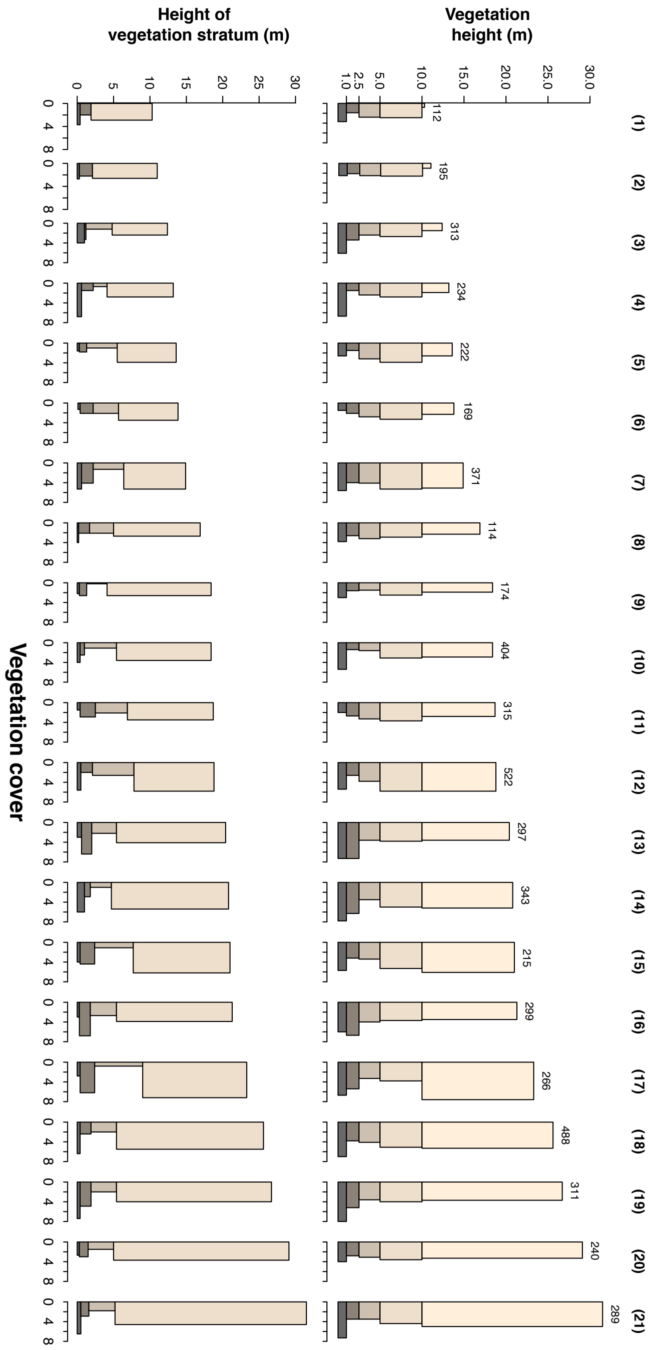

Figure S2 Vertical profiles of woodland and forest vegetation at our 21 sites across eastern Australia. Bar widths depict cover (0–10) in both *height strata* (upper panel) and *vegetation strata* (lower panel). Height strata were defined by five fixed height bands (0–1 m, 1.1–2.0 m, 2.1–5 m, 5.1–10 m, >10 m), while vegetation strata were defined as four vegetation layers, namely herbaceous, shrub, subcanopy, and canopy. The sites are ordered from left to right according to their mean canopy height (from lowest to highest) as in Table S1. The numbers just above the bars in the upper panel show the number of prey attacks recorded at the site. The numbers in parentheses along the top of the figure are site codes as in Figure S1 and Table S1.

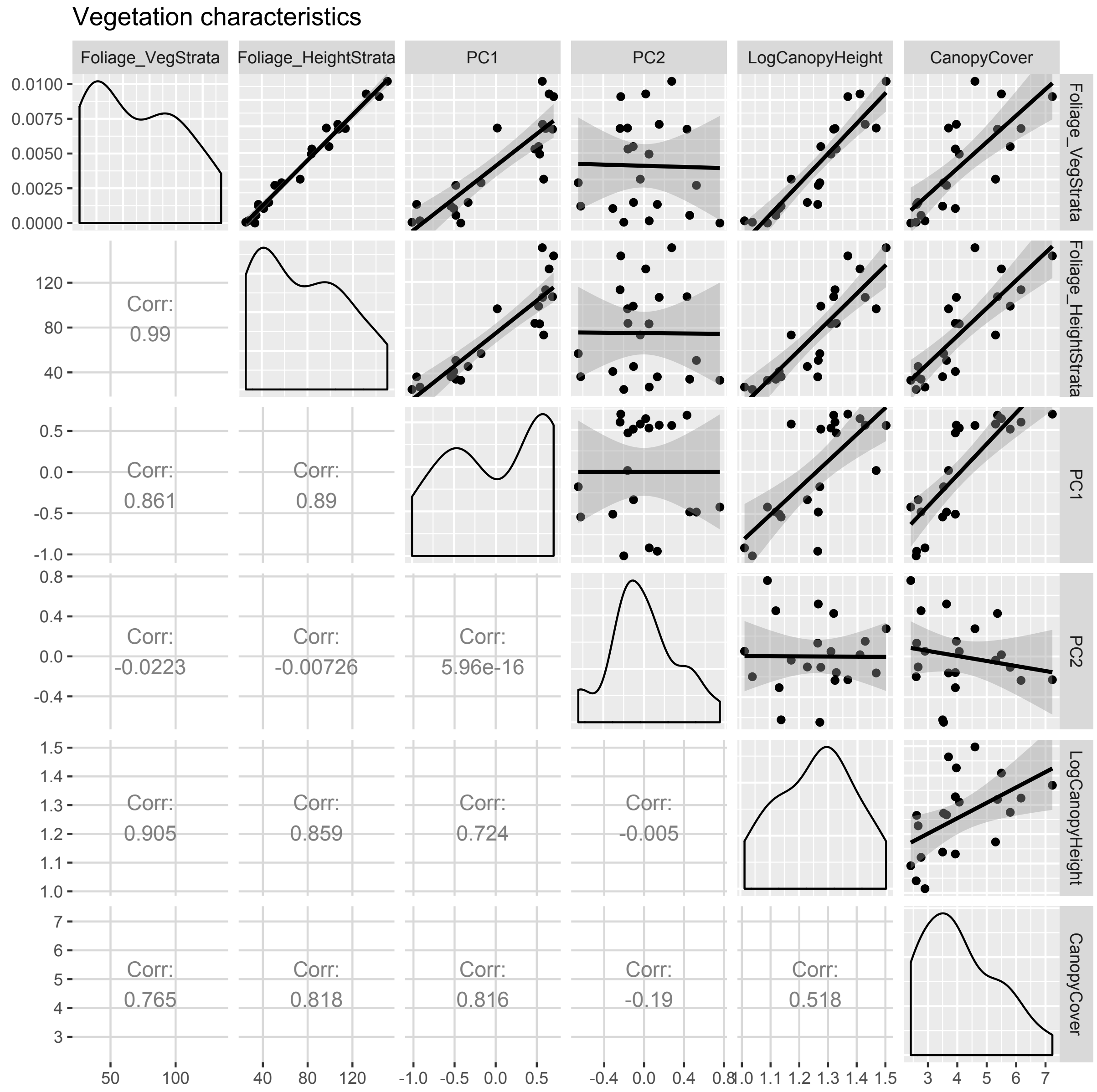

Figure S3 Relationships between vegetation characteristics across our 21 study sites. "Foliage_VegStrata" is the sum of foliage coverage of all *vegetation strata* at each site. "Foliage_HeightStrata" is the same for *height strata*. For the definition of *vegetation strata* and *height strata*, please see Appendix A1. PC1 and PC2 are the first two PC axes from a PCA analysis on all vegetation characteristics (see Fig. S4).

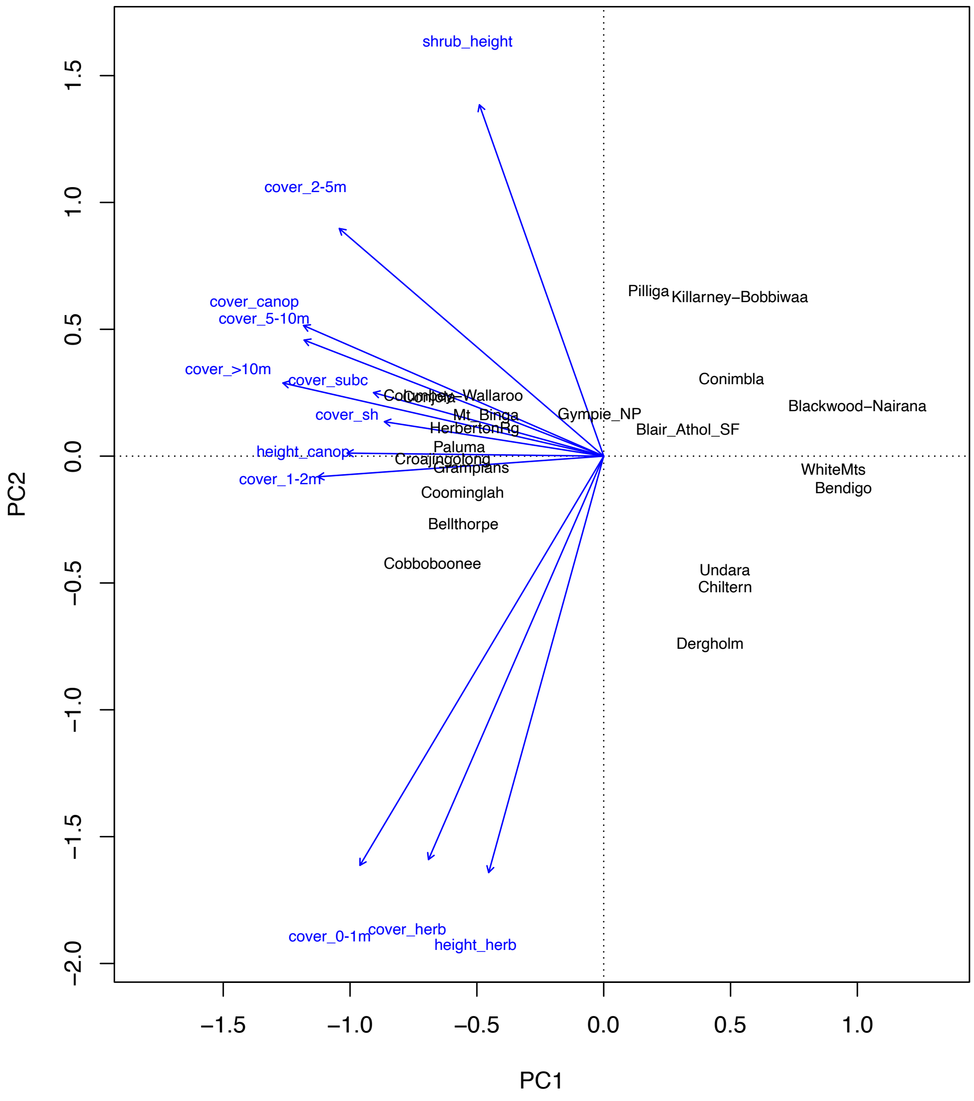

Figure S4 The first two PC axes from a PCA run on all vegetation characteristics. Most vegetation characteristics were positively correlated with PC1 (48% of variability explained). The exception was the ground layer (cover_0-1m = coverage 0-1 m, cover_herb = coverage of herbs, height_herb = herb height), which correlated with PC2 (16% of variability). Shrub height (shrub_height) was also correlated more with PC2 than PC1. Further labels are as follows: cover_2-5m = coverage 2-5 m, cover_canop = coverage of canopy, cover_5-10m = coverage 5-10 m, cover_>10m = coverage over 10 m, cover_subc = coverage of subcanopy, cover_sh = coverage of shrubs, height_canop = canopy height, cover_1-2m = coverage 1-2 m.
